# Chemical and Biological Properties of *S*-1-Propenyl-l-Cysteine in Aged Garlic Extract

**DOI:** 10.3390/molecules22040570

**Published:** 2017-03-31

**Authors:** Yukihiro Kodera, Mitsuyasu Ushijima, Hirotaka Amano, Jun-ichiro Suzuki, Toshiaki Matsutomo

**Affiliations:** Drug Discovery Laboratory, Wakunaga Pharmaceutical Co., Ltd., Hiroshima 739-1195, Japan; ushijima_m@wakunaga.co.jp (M.U.); amano_h@wakunaga.co.jp (H.A.); suzuki_j@wakunaga.co.jp (J.-i.S.); matsutomo_t@wakunaga.co.jp (T.M.)

**Keywords:** garlic (*Allium sativum* L.), *S*-1-propenyl-l-cysteine, processing, aged garlic extract, organic synthesis, isomerization, immunomodulatory effect, antihypertension, pharmacokinetics, drug-drug interactions

## Abstract

*S*-1-propenyl-l-cysteine (S1PC) is a stereoisomer of *S*-allyl-l-cysteine (SAC), an important sulfur-containing amino acid that plays a role for the beneficial pharmacological effects of aged garlic extract (AGE). The existence of S1PC in garlic preparations has been known since the 1960’s. However, there was no report regarding the biological and/or pharmacological activity of S1PC until 2016. Recently, we performed a series of studies to examine the chemical, biological, pharmacological and pharmacokinetic properties of S1PC, and obtained some interesting results. S1PC existed only in trace amounts in raw garlic, but its concentration increased almost up to the level similar of SAC through aging process of AGE. S1PC showed immunomodulatory effects in vitro and in vivo, and reduced blood pressure in a hypertensive animal model. A pharmacokinetic study revealed that S1PC was readily absorbed after oral administration in rats and dogs with bioavailability of 88–100%. Additionally, S1PC had little inhibitory influence on human cytochrome P450 activities, even at a concentration of 1 mM. Based on these findings, S1PC was suggested to be another important, pharmacologically active and safe component of AGE similar to SAC. In this review, we highlight some results from recent studies on S1PC and discuss the potential medicinal value of S1PC.

## 1. Introduction

Several plants belonging to the genus *Allium* have been historically used by humans. Among these plants, garlic (*Allium sativum* L.) has been widely recognized to possess medicinal properties against several diseases or symptoms during the time when other medicinal supplies were limited [1]. Many people in the world still use garlic and its preparations for their health benefits, as several scientific studies have reported its following properties; antioxidative, antithrombotic, hypolipidemic, hypoglycemic, antihypertensive, and anti-Alzheimer’s disease effects [2,3]. Earlier, people used garlic as a remedy without knowledge of scientific evidence [1,4]. The advancement of modern science has revealed the chemical and biological properties of various constituents in garlic and its preparations. After the noteworthy discovery of the allicin and allicin–allinase system in the early 1940’s, the studies on chemical properies of garlic have rapidly advanced [1,4,5]. Many studies have focused on evaluation of allicin and its transformed chemicals, considering these compounds as beneficial constituents for our health in garlic and its many preparations [6]. Various reports regarding the beneficial biological activities of allicin, such as antimicrobial effect, antioxidant effect, suppression of cholesterol biosynthesis, anticancer activity and anti-inflammation effect, have emerged since its discovery ([1,5,6]). However, unfavorable chemical properties of allicin, such as high reactivity and instability, were also revealed [7,8], raising questions regarding allicin being really an active, beneficial component of garlic [1,7,8,9].

**Figure 1:**
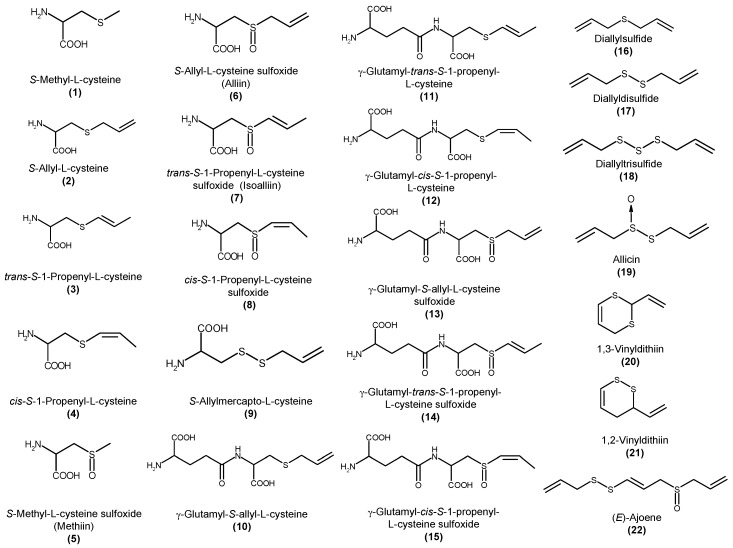
Chemical structures of sulfur-containing compounds derived from garlic.

Researchers have been also interested in hydrophilic compounds, such as methiin (*S*-methyl-l-cysteine sulfoxide), allicin (*S*-allyl-l-cysteine sulfoxide), *S*-methyl-l-cysteine (SMC), *S*-allyl-l-cysteine (SAC), *S*-allylmercapto-l-cysteine (SAMC) and γ-glutamyl-*S*-alkenyl-l-cysteines (Figure 1) [1,5,9]. Among these hydrophilic compounds, SAC has been extensively studied and shown to possess various biological activities, such as anticancer, antioxidant, cholesterol-lowering, anti-hepatotoxic, and neuroprotective effects [1,9,10]. SAC has also been closely examined for its pharmacokinetic, safety and physical and chemical properties [11,12]. *S*-1-propenyl-l-cysteine (S1PC), a stereoisomer of SAC, was found to be present in garlic in the early 1960s [13]. Since its discovery, there have been several reports on organic synthesis methods to obtain S1PC as an intermediate compound in the synthesis of *trans*-*S*-1-propenyl-l-cysteine sulfoxide (*trans*-S1PCSO, isoallicin), which is a precursor of the lachrymatory compound in onion [14,15,16,17]. However, there were no reports concerning the biological activities of S1PC until 2016. Recent studies suggest that S1PC has unique chemical properties [18,19], and it may partly contribute to the biological activities of garlic preparations in immunomodulation and cardiovascular diseases [20,21].

Recently, studies assessing the effectiveness and safety of natural products as food/supplements and medical agents showed that their concomitant use sometimes caused serious undesirable effects on human health [22,23,24,25,26,27]. Furthermore, pharmacokinetic studies are also important to evaluate biological activities of the constituents of natural products because the active constituents must be fully absorbed and sufficiently distributed to the target organs or tissues for their pharmacological effects. S1PC has been shown to be orally well absorbed with little inhibitory effect on human cytochrome P450 (CYP) activity in vitro [28,29].

In this review, we describe the chemical and biological properties of S1PC and discuss its medicinal potential by reviewing recent studies on its chemical synthesis, biosynthesis, production mechanism in garlic preparation, analytical procedure, biological and pharmacologic effects, pharmacokinetics and safety.

## 2. Organic Synthesis of *trans*-*S*-1-propenyl-l-cysteine

(*R*)-3-[(*E*)-Prop-1-enylsulfinyl]-2-aminopropanoic acid, an odorless sulfur-containing amino acid referred to as *trans*-S1PCSO or isoalliin, has been isolated from onion (*Allium cepa* L.) and confirmed to be a precursor of the lachrymatory compound, a biologically active compound that is beneficial for human health [5,16,17,30,31]. The content of this compound in onion is less than 0.2% [16], and its purification is difficult because many compounds with similar chemical characteristics exist in onion. Therefore, its organic synthesis method has been investigated to reveal sulfur chemistry in onion and other *Allium* vegetables, including the preparation of S1PC as an intermediate of S1PCSO [14,15,16,17]. S1PC has two forms of isomers, *trans*-*S*-1-propenyl-l-cysteine (*trans*-S1PC, (**3**)) and *cis*-*S*-1-propenyl-l-cysteine (*cis*-S1PC, (**4**)) (Figure 1) [15,18]. Since the *trans*-form of S1PC, oxidized *trans*-S1PC (isoalliin) and their γ-glutamyl derivatives are naturally present in *Allium* plants [13,14,32], researchers have focused on the stereoselective synthesis of *trans*-form S1PC.

**Scheme 1:**
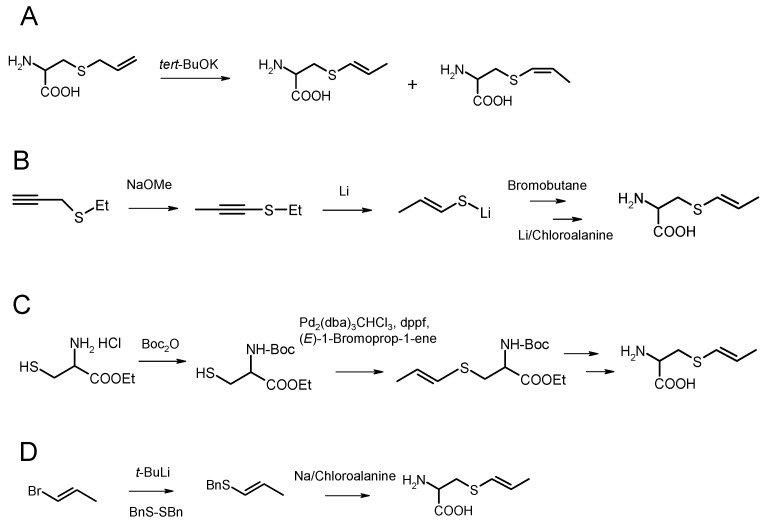
Organic syntheses of *S*-1-propenyl-l-cysteine. Reaction schemes **A**–**D** were redrawn based on previous studies [14,15,16,17].

In the mid 1960s, Carson and Boggs reported a simple and facile method to obtain S1PC by the isomerization of SAC under the strong basic condition using potassium *tert*-butoxide (Scheme 1A) [14]. This method yielded both *cis*- and *trans*-S1PC in almost equal amounts, and the *cis*-form was mainly recovered from the concentrated aqueous solution. Although this method is very simple and easy to perform, some additional purification steps, such as chromatographic separations, are necessary to obtain the *trans*-isomer with high purity. Additionally, there is a possibility that the strong basic conditions used in this method induce the racemization reaction [33,34,35]. Nishimura et al. successfully obtained *trans*-S1PC in a 5-step process involving isomerization of a terminal triple bond ethyl prop-2-ynyl sulfide to ethyl prop-1-ynyl sulfide and the subsequent reductive coupling with alkyl chloride under strong basic conditions using sodium methoxide, metal lithium and liquid ammonia (Scheme 1B) [15]. Approximately three decades after Nishimura’s report, Namyslo and Stanitzek reported another synthesis method for *trans*-S1PC, utilizing special palladium–catalyzed coupling of cysteine with (*E*)-1-bromoprop-1-ene (Scheme 1C) [16]. Their method consisted of four steps, each showing a relatively high yield, 98%, 82%, 94% and 78% (final calculated yield: 59%), respectively. Lee et al. also used (*E*)-1-bromoprop-1-ene to synthesize S1PC stereoselectively (Scheme 1D) [17]. They obtained (*E*)-1-(benzylthio)-1-propene by treating *tert*-BuLi and dibenzyl disulfide with (*E*)-1-bromoprop-1-ene under strong basic conditions using metal sodium and liquid ammonia, and subsequently coupled with 3-chloroalanine to produce *trans*-S1PC. Although their method required only two steps, the yield of each step was 48% and 27% (final yield calculated: 13%). Kodera et al. observed reversible isomerization of S1PC under acidic conditions [18], suggesting that *trans*-S1PC prepared by the above method consisted of a mixture of *cis*- and *trans*-forms resulting from the removal of protective groups from amino and/or carboxylic groups. Therefore, further study must be made to accomplish the sufficient stereoselective synthesis of *trans*-S1PC including the purification steps.

## 3. Biosynthesis of *S*-1-propenyl-l-cysteine in *Allium* Plants

**Scheme 2:**
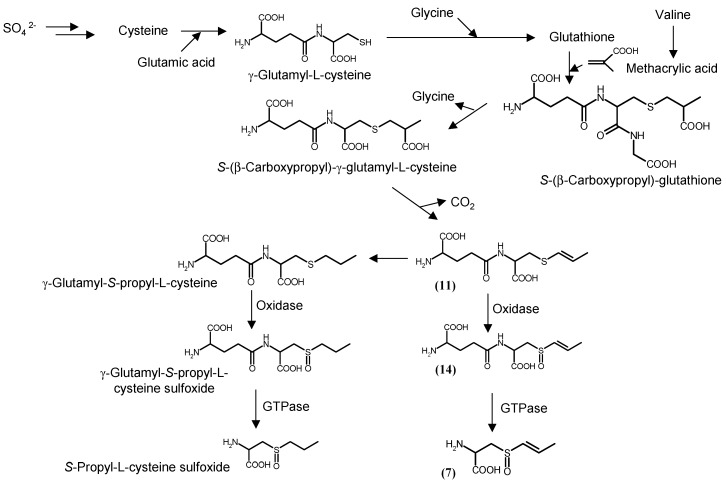
Biosynthesis of sulfur-containing compounds in onion. Isoalliin (**7**) biosynthesis pathway was presented by Parry et al. using an isotope–labeled compound in onion [40,41]. Scheme of biosynthesis was redrawn previous reports ([1], pp. 41–46, pp. 68–70, pp. 74–76, [5], pp. 165–172). The chemical names of the numbered compounds are shown in Figure 1.

**Scheme 3:**
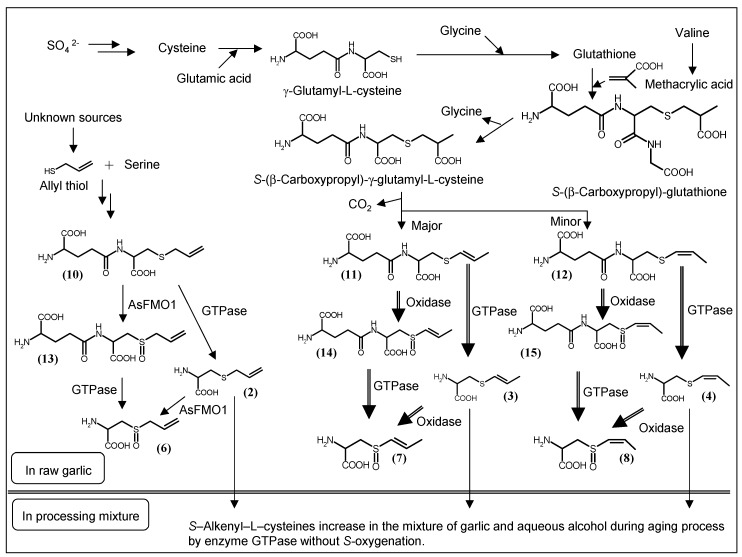
Biosynthesis of sulfur-containing compounds in garlic. The biosynthesis pathway of alliin (**6**) in garlic was presented by Yoshimoto et al., who identified a flavine-containing monooxygenase (AsFMO1) [38,39]. Isoalliin biosynthesis was presented by Parry et al. using an isotope-labeled compound in onion [40,41]. Isoalliin (**7**) content in garlic is less than 10% of the alliin content, and *trans*-GS1PC (**11**) content is high without the *S*-oxygenation reaction. It remains to be examined whether AsFMO1, an *S*-oxidase identified in garlic, *S*-oxygenates S1PCs and/or GS1PCs to S1PCSOs and/or GS1PCSOs in raw garlic. When garlic is processed in aqueous alcohol, SAC and S1PCs contents increased in the processing mixture without *S*-oxygenation. Illustration is based on mechanisms published in previous reports ([1,5,38,39]). Double line arrows indicate putative reactions based on the alliin biosynthesis pathway reported by Yoshimoto et al. [38,39]. The chemical names of the numbered compounds are shown in Figure 1.

*Allium* plants accumulate organic sulfur compounds biosynthetically using the ultimate inorganic source, sulfate (SO_4_^2^^−^) (Scheme 2 and Scheme 3) [1,5]. Sulfur is incorporated into l-cysteine, which subsequently undergoes two conversions, glutamylation and glycylation, to yield glutathione. After this sulfur fixation, various sulfur compounds are produced as a series of sulfur storage molecules, such as *S*-alk(en)yl-l-cysteine sulfoxides, γ-glutamyl-*S*-alk(en)yl-l-cysteines and γ-glutamyl-*S*-alk(en)yl-l-cysteine sulfoxides via *S*-alk(en)ylation of l-cysteine residue, followed by removal of glycine to form γ-glutamylpeptides and by *S*-oxygenation and deglutamylation to yield *S*-alk(en)yl-l-cysteine sulfoxides [1,5,36,37,38,39]. Four *S*-alk(en)yl-l-cysteine sulfoxides have been identified in *Allium* (Figure 1) and the relative abundance levels of these compounds are different among species. Parry et al. presented the biosynthesis pathway of *S*-1-propenyl compounds including *trans*-S1PCSO (isoalliin) (7) in onion, through isotope-labeling studies [5] and [40,41]. In the onion, after decarboxylation of the β-carboxypropenyl residue in *S*-(β-carboxypropenyl)-γ-glutamyl-l-cysteine to yield γ-glutamyl-*S*-1-propenyl-l-cysteine (GS1PC), sulfur in GS1PC is oxidized by oxygenase and subsequently isoalliin is released from GS1PC sulfoxide by deglutamylation (Scheme 2).

Garlic contains SAC (2) and its putative precursor compound, γ-glutamyl-*S*-allyl-l-cysteine (GSAC, (**10**)) [1,18,19]. However, the source of the allyl group in *S*-alkenylation and the reaction order between *S*-oxidation and deglutamylation to produce alliin is unclear [38,39]. Flavin-containing monooxygenase (FMO) is widely distributed in plants and plays a critical role in oxidation reactions during the biosynthesis [42]. Yoshimoto et al. showed that garlic flavin-containing monooxygenase (AsFMO1) catalyzes the highly stereoselective *S*-oxygenation reaction in the biosynthesis of alliin in garlic [38,39]. Recombinant AsFMO1 catalyzed *S*-oxygenation of both SAC and GSAC, but strongly preferred SAC over GSAC as the *S*-oxidation substrate. The content of SAC in fresh garlic is less than 0.026 mg/g fresh-weight [1]. Therefore, these results suggested that alliin was produced via rapid *S*-oxidation of SAC by AsFMO1 after deglutamylation of GSAC in garlic (Scheme 3).

Garlic contains both isoalliin and GS1PC, and the order of reaction between *S*-oxidation and deglutamylation is unclear. Herein, the amount of S1PC, a deoxidized form of isoalliin, is less than the limit of detection in fresh garlic cloves, no more than 0.006 mg/g fresh-weight in crushed garlic, while isoalliin exists in the range of 0.2–1.2 mg/g fresh-weight in garlic [1]. However, the content of GS1PC, a precursor of S1PC, is 3–9 mg/g fresh-weight, which is more than that of GSAC (2–6 mg/g fresh-weight) [1]. GS1PC is abundant as a sulfur storage molecule in raw garlic without undergoing two conversions: *S*-oxygenation and deglutamylation. S1PC and GS1PC were identified in aged garlic extract [1,18,19], and the content of GS1PC in AGE was around 5% of that in raw garlic [1,18,32], suggesting that the deglutamylation reaction without *S*-oxygenation may hardly occur in raw garlic, whereas only deglutamylation of GS1PC occurs during the aging process in aqueous solution (Scheme 3). According to the above findings, isoalliin in raw garlic may be produced by the same synthetic pathway as alliin [38,39], and S1PC in AGE is produced by the same production mechanism as SAC [1,9,19].

## 4. Analysis of Sulfur-Containing Compounds in Garlic and Its Preparations

*Allium* plants and their preparations contain a variety of characteristic sulfur-containing compounds. These compounds are categorized as hydrophilic and hydrophobic. Gas chromatographic (GC) methods have outstanding separation abilities and are generally used for the analysis of hydrophobic compounds and the volatile derivatives of hydrophilic compounds. There are numerous reports describing the analysis of volatile compounds derived from *Allium* plants [43,44,45,46]. During GC analysis, pyrolysis of analytes and formation of degradation products during the measurement must be considered, as the analytes are often exposed to high temperatures at the inlet port and in the GC-column oven of the analytical equipment. Decomposition by heat resulted in peaks of artifacts [47,48,49].

High performance liquid chromatography (HPLC) has been widely used for the analysis of both hydrophilic and hydrophobic compounds. HPLC analysis is usually run at relatively low temperatures to avoid decomposition [50]. Additionally, many substances with various chemical properties can be analyzed in a single run using a gradient program, and/or changing the content of organic solvent in mobile phase. For these reasons, HPLC methods are extremely useful for the analysis of natural products. However, the problem of interference by co-existing components in a sample often occurs because of the use of simple UV absorption-based detection. Lawson et al. presented an HPLC method for S1PC analysis at UV 220 nm absorption. However, the peak corresponding to S1PC was partially obstructed by other components in a garlic sample incubated with GTPase [32]. Yamasaki et al. successfully separated eleven cysteine derivatives including alliin, isoalliin and deoxyalliin (SAC) in a garlic sample using a reversed phase HPLC method at UV 210 nm absorption, but S1PC was not observed in their report [51]. In fact, many researchers faced problem of interference by co-existing components under separation conditions used for analysis of hydrophilic compounds in a sample [52,53,54,55,56,57]. Therefore, an analytical method specific for the detection of sulfur-containing compounds was necessary.

**Figure 2:**
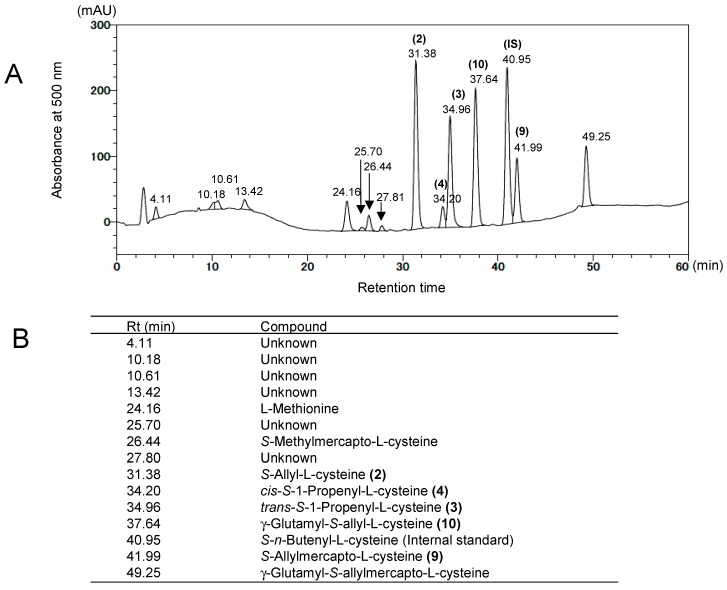
Chromatogram of hydrophilic sulfur compounds in aged garlic extract by sulfur specific post-column HPLC analysis using hexaiodoplatinate reagent. Chromatogram was monitored at 500 nm absorbance. HPLC analysis was performed by post-column HPLC method using a NEXERA X2 system (Shimadzu, Kyoto, Japan) according to a previous report [19]. (**A**) Chromatogram of aged garlic extract; (**B**) Identified compounds and their elution times on HPLC chromatogram. The chemical names of the numbered compounds are shown in Figure 1.

Hexaiodoplatinate reagent (HIPR) is a dark red substance whose color changes to yellow when mixed with sulfur compounds [57,58,59,60]. Matsutomo et al. successfully developed a post-column HPLC method for the detection of sulfur-containing compounds by using HIPR [19,59,60]. They developed the method to separate and analyze more than twenty sulfur-containing compounds in garlic preparations, including isomers of S1PC ((**3**) and (**4**) in Figure 2A), in a single run. Additionally, this method was adapted for LC-MS analysis, using a separating mobile phase consisting of an organic solvent, methanol, and other reagents, formic acid or heptafluorobutyric acid, which do not have undesirable influences on the MS system. *S*-*n*-Butenyl-l-cysteine, which does not exist in the genus *Allium*, was used as the internal standard for both HPLC and LC–MS analysis (eluted at 40.95 min in Figure 2A,B). Using this method, *cis*-form S1PC was shown to exist in AGE [19], and its content increased during the aging process of AGE [18,19]. The authors concluded that the established HPLC method may help identify unknown sulfur-containing compounds in natural products and could be utilized for elucidating of their production mechanism.

## 5. Change of *S*-1-propenyl-l-cysteine Content during Processing

**Figure 3:**
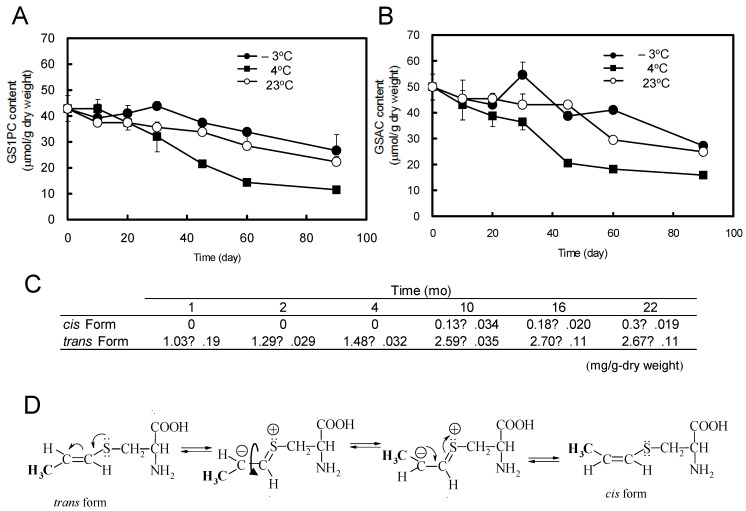
Changes of sulfur-containing compounds in raw garlic and AGE. (**A**) Changes of *trans*-GS1PC content in raw garlic stored at −3 °C, 4 °C and 23 °C; (**B**) Changes of GSAC content in raw garlic stored at −3 °C, 4 °C and 23 °C; (**C**) Changes of *trans*-S1PC and *cis*-S1PC content in aged garlic extract during the aging period. Values are mean ± SD (*n* = 3); (**D**) Putative production mechanism of *cis*-S1PC through isomerization of the *trans*-form during aging process. Illustration is drawn based on literatures reports [18,19,53].

Garlic contains several γ-glutamylpeptides, such as γ-glutamyl-*S*-methyl-l-cysteine (GSMC), GSAC and GS1PC [1,5,32,37,51,57]. It was observed that the amount of these peptides decreased when garlic was soaked in aqueous alcohol, while the formation of sulfur-containing amino acids, such as SMC, SAC and S1PC increased [1,9,18,19]. This change was shown to occur through hydrolysis of γ-glutamylpeptides by GTPase [1,39]. It was also shown that this hydrolysis reaction was complete within 100 days at room temperature [1]. The amount of S1PC in the garlic preparations varied depending on the content of its precursor compound, GS1PC, in raw garlic (3–9 mg/g fresh-weight) [1]. Ichikawa et al. revealed an interesting phenomenon related to the content of GS1PC in fresh garlic (Figure 3A,B). They stored fresh garlic at −3 °C, 4 °C and 23 °C for 150 days, and measured the content of GS1PC at an appropriate interval. The amount of GS1PC decreased markedly between 10 and 60 days of storage at −3 °C, 4 °C and 23 °C, to 88%, 34% and 75% (on a molar basis) of the initial content, respectively [53]. Similar results were observed in the GSAC content during the storage of fresh garlic under the same conditions, suggesting that the common temperature of refrigeration, 4 °C, might not be suitable to prevent the transformation of γ-glutamylpeptides in fresh garlic (Figure 3A, B).

Although *trans*-S1PC is naturally present in garlic, *cis*-S1PC is also present in AGE [19]. Interestingly, the amount of *cis*-S1PC in AGE gradually increased during the aging period, while that of *trans*-S1PC became constant after 10 months of aging (Figure 3C) [19]. The presence of *cis*-GS1PC, a plausible precursor of *cis*-S1PC, was confirmed in raw garlic (Scheme 3) [1,32]. However, its amount was not enough to produce the *cis*-S1PC content detected in AGE [18,32]. In order to explain this unbalanced stoichiometry, Kodera et al. performed the model reaction to verify their hypothesis that *cis*-S1PC is produced by isomerization of *trans*-S1PC during the aging period [18]. In their study, *cis*-S1PC and *trans*-S1PC were dissolved in deuterium oxide separately, and acetic acid-*d*_4_ was added to each solution to adjust the pH between 3 and 5. The solutions were dispensed into NMR tubes, incubated at 60 °C for 30 days and analyzed using ^1^H-NMR at appropriate intervals. The proton signals of C1, C2 and C3 in the propenyl group of the incubation mixtures changed gradually, until the intensities of *cis*- and *trans*-signals were almost equal. These results indicated that isomerization of S1PC between the *cis*-form and *trans*-form is reversible, and the amount of *cis*-S1PC observed in AGE might be partly derived from *trans*-S1PC through the isomerization reaction during the aging process (Figure 3D). This may be one of the reactions occurring during aging, producing the variety of characteristic constituents of AGE.

## 6. Biological Properties of *S*-1-propenyl-l-cysteine 

The variety of biological properties of SAC, a stereoisomer of S1PC, such as anti-cancer effects, antioxidant effect, neuroprotection, cholesterol reduction may be important for maintenance of human health [3,9,10]. Furthermore, SAC shows an excellent pharmacokinetic profile with high bioavailability and safety [11,12]. S1PC was identified in AGE, and its content was similar to that of SAC [18,19]. However, there was no information regarding the biological effects, pharmacokinetics and safety of S1PC. Herein, we will describe the biological properties of S1PC that have been revealed by recent studies [20,21,29,30].

### 6.1. Immunomodulation

Garlic has been speculated to possess immunomodulatory effects. Immunomodulation by several garlic preparations and many sulfur-containing compounds has been studied using various in vitro and in vivo experimental systems. Allicin, diallylpolysulfides (DAS*_n_*, *n* = 1–7), allyl methyl sulfide, vinyldithiins and ajoenes are representative hydrophobic compounds derived from garlic (Figure 1). Dirsch et al. showed that allicin and ajoene reduced accumulation of nitric oxide (NO) through inhibition of inducible nitric oxide synthase (iNOS) activity in LPS-stimulated RAW 264.7 macrophages [61]. Kang et al. observed that allicin induced tumoricidal activity along with enhanced formation of TNF-α and NO in a dose-dependent manner (1–100 ng/mL) in murine peritoneal macrophages [62]. Oral administration of allicin enhanced the formation of pro-inflammatory mediators IFN-γ, TNF-α and IL-12p70 in BALB/c mice [63]. In vitro studies revealed that other hydrophobic compounds, including DAS (**16**), DADS (**17**) and DATS (**18**), which are major transformed compounds of allicin, showed reduction of iNOS expression/activity and NO production as well as inhibition of inflammatory cytokine production and inhibition of NF-κB activity [64,65,66].

On contrary, alliin, a precursor of allicin in raw garlic, was the only hydrophilic compound that was studied to assess its anti-inflammatory activity. Alliin prevented an increase in the expression of pro-inflammatory genes *IL*-*6*, *MCP*-*1* and *Erg*-*1* and in the protein levels of IL-6 and MCP-1 under LPS stimulation in 3T3-L1 adipocytes [67]. Alliin also produced an increase in IL-1β and TNF-α levels in peripheral blood mononuclear cells during pokewood mitogen stimulation [68]. Although the characteristic compounds in raw garlic mentioned above showed anti-inflammatory effects at low concentrations in in vitro experiments, they also induced or produced pro-inflammatory mediators. Additionally, these compounds quickly disappeared when they were mixed with red blood cells [7,8]. Therefore, more comprehensive studies of these compounds, including pharmacokinetic studies, are necessary to evaluate the activity of raw garlic.

**Figure 4:**
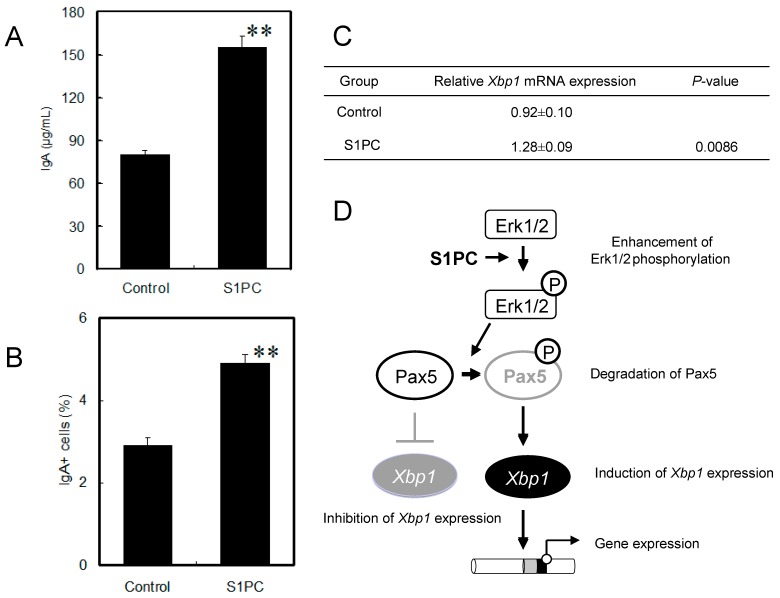
Immunomodulatory effect of *S*-1-propenyl-l-cysteine. (**A**) Effect of S1PC on IgA levels in intestinal lavage fluid. S1PC (30 mg/kg, once a day) or distilled water were orally administrated to C57BL/6N mice for 5 days (*n* = 4–5). IgA level was measured using Mouse IgA ELISA Quantification set (Bethyl Laboratories, Inc. Montgomery, AL, USA), (** *p* < 0.01); (**B**) Effect of S1PC on IgA–inducing cells in Peyer’s patches (PPs). The population of IgA+ cells was analyzed on a FACSCalibur (BD Bioscience, Palo Alto, CA, USA) using the DB Cell Quest software, (** *p* < 0.01); (**C**) Effect of S1PC on *Xbp1* mRNA expression in PP. Quantitative real–time PCR reactions were performed using a Piko Real Real–time PCR system (Thermo Fisher Scentific, Waltham, MA, USA) to determine relative expression levels of target genes. *p*-Values identified statistically significant differences between the test and control groups using a Student’s *t*-test; (**D**) Putative mechanism on induction of *Xbp1* expression by S1PC. S1PC enhances the phosphorylation of Erk1/2 and the degradation of Pax5, thereby increasing the expression of *Xbp1* mRNA. *Xbp1* induces the expression of several genes. Illustration based on a literature report [20].

AGE has been reported to have immunostimulatory effects in animal studies and human clinical trials [69,70,71,72]. However, its active constituents have not been identified. Suzuki et al. observed that S1PC increased immunoglobulin A (IgA) production by increasing the number of IgA-producing cells in vitro and in vivo (Figure 4A,B) [20]. In addition, S1PC increased the mRNA expression of *X-box binding protein* *1* (*Xbp1*), an inducer of plasma cell differentiation, by enhancing Erk1/2-mediated paired box protein 5 (Pax5) degradation in B cells (Figure 4C,D). IgA plays an important role in the first line of immune defense by the intestinal mucosa and is produced by plasma cells in gut–associated lymphoid tissues [73,74,75,76]. Therefore, S1PC may enhance the intestinal immune response against pathogens.

AGE has been reported to activate natural killer (NK) cells and subsequently inhibit tumor growth in experimental animal and human clinical studies [69,70,71]. The activation of NK cells is known to regulated by type I IFNs [77,78,79]. Recently, the transcription factor *Xbp1* has been shown to be essential for the development, survival and cytokine production of several immune cells. Dendritic cells (DC) from *Xbp1* knockout mice and macrophages decrease the production of type I IFNs [80,81]. In addition, induction of the unfolded protein response (UPR) and splicing of *Xbp1*s are essential for optimal NK cell cytotoxicity [82]. Futhermore, the phosphorylation of Erk1/2 increases NK cell cytotoxicity [83]. Suzuki et al. observed that S1PC increased the phosphorylation of Erk1/2 and *Xbp1* mRNA expression [20]. Therefore, S1PC may affect the modulation of NK cell and DC via the activation of Erk1/2, the up–regulation of *Xbp1* expression and subsequent signaling pathways, and thus eventually may bolster human immunes responses.

### 6.2. Antihypertension

Lifestyle and diet strongly influence human health. The number of people who contract lifestyle-related diseases, such as diabetes, hyperlipidemia, hyperglycemia and hypertension, is increasing world-wide [84]. Hypertension is an important risk factor for cardiovascular diseases (CVD), and is one of the most frequently encountered health problems [85,86]. The effect of garlic supplementation on blood pressure has been examined in numerous clinical studies [87]. Meta-analysis of 20 clinical trials performed in 1988–2013 revealed that garlic supplements reduced the blood pressure of 970 participants with a mean ± SE decrease in SBP of 5.1 ± 2.2 mm Hg (*p* < 0.001) and a mean ± SE decrease in DBP of 2.5 ± 1.6 mm Hg (*p* < 0.002) [87]. The supplements tested in these trials were produced from dehydrated raw garlic powder, garlic oil, AGE, and Japanese garlic powder containing egg yolk. Ried et al. evaluated the antihypertensive effect of AGE on adult patients with uncontrolled hypertension in double–blind randomized placebo-controlled parallel trials. In those studies, AGE was consumed for 12 weeks, and the blood pressure-lowering effects were observed after 8 weeks of AGE treatment with significant differences between the placebo and AGE groups. They concluded that AGE consumption may be effective, tolerable, and safe adjunct treatment to conventional antihypertensive therapy [88,89].

Spontaneously hypertensive rats (SHRs) have been used as an experimental animal model for human essential hypertension, although the exact pathogenic mechanism of hypertension in SHRs is still unclear [90,91]. Comparison of the antihypertensive effects of raw garlic and AGE on SHRs showed that the effects in the raw garlic group were noticeably stronger than those in animals receiving AGE. However, the raw garlic group showed serious side effects including anemia, an induction of body weight loss and a decrease in the number of red blood cells, while those undesirable effects were not observed in the AGE group [22]. Several constituents have been considered to be responsible for the antihypertensive effects of garlic supplements, including alliin, allicin and SAC. However, detailed mechanisms of their actions are unclear. Alliin and allicin are representative compounds in fresh garlic, but their pharmacokinetic studies revealed that the bioavailability of alliin was quite low, 16.5% [92], and allicin was highly unstable in the both rats and human blood [7,8]. 85.5% of ^35^S-labeled allicin was excreted in urine and feces in rats [93], suggesting that both compounds would not be the real active components. On the contrary, SAC showed excellent oral absorption in experimental animals with a bioavailability of almost 100% [11]. Concentration–time profiles of SAC in human plasma were similar to those observed in mice, rats and dogs (rapid absorption and long-lasting in blood concentration after intake of AGE) [11,12]. The effectiveness of SAC and AGE were evaluated in a hypertensive animal model, 5/6 nephrectomized rats. Treatment with SAC (200 mg/kg, i.p.) and AGE (1.2 mL/kg, i.p.) for 30 days ameliorated hypertension, as well as other indicators of renal impairment [94].

Matsutomo et al. studied and compared the effects of AGE and its two major sulfur-containing constituents, SAC and S1PC, on the development of hypertension in SHRs, which were orally administrated AGE (2 g/kg including 7.9 mg/kg of SAC and 6.5 mg/kg of S1PC), SAC (7.9 mg/kg) and S1PC (6.5 mg/kg) for 10 weeks, respectively [21]. AGE and S1PC significantly inhibited the increase of SBP in SHRs at 10 weeks of treatment. The development of hypertension was delayed in the SAC treated animals, but was not significantly different from the vehicle-treated controls. Metabolomics analyses of plasma samples collected from SHRs after completion of the treatment showed that plasma concentrations of seven metabolites were affected by the treatment of S1PC (Table 1). Furthermore, regression analysis showed the correlation between SBP and a plasma levels of betaine, tryptophan and LysoPCs. Betaine is known as an organic osmolyte in renal medullary cells, and its concentration in plasma and urine is controlled by homeostatic system [95]. Thus, the reduction of betaine in the S1PC treatment group may be partially related to the antihypertensive effect of S1PC. Matsutomo et al. suggested that S1PC lowers the blood pressure through alteration of plasma tryptophan levels via the kynurenine pathway, which leads to the reduction of blood pressure, though clear evidence of metabolite changes in these pathways was not obtained [21,96]. Altered concentrations of phospolipids were also observed in the group receiving S1PC treatment. LysoPC is known to affect vasorelaxation as well as NO production [97]. These results suggest that S1PC reduces the blood pressure through alteration of metabolite levels in the pathway involving glycerolipid metabolism, tryptophan metabolism, glycine metabolism, and serine and threonine metabolism, and may serve as one of the major active constituents responsible for the antihypertensive effect in AGE.

### 6.3. Pharmacokinetics and Safety

In assessing the biological and pharmacologic activities of constituents of garlic and its preparations in vivo, we must consider their pharmacokinetic profiles, which are influenced by physiological functions of the body associated with oral absorption, tissue distribution, metabolism, and excretion. To produce a pharmacologic effect, each active constituent must be sufficiently absorbed and reach the target organs or tissues. Several studies examined the pharmacokinetics of hydrophobic sulfur components from garlic preparations, such as allicin, vinyldithiins, and allylpolysulfides, in mice and rats after oral administration [93,98,99,100]. The results of the studies showed that those hydrophobic compounds were absorbed in the gastrointestinal tract, but were not detected in rodent urine samples, due to their metabolic instability. The pharmacokinetics of several hydrophilic components in garlic preparations, such as alliin, SAC, and cycloalliin, were also studied [11,12,53,92,101]. Guo et al. reported that the oral bioavailability of alliin, a precursor compound of allicin, was 16.5% in rats [92]. Ichikawa et al. evaluated the pharmacokinetics of cycloalliin a constituent in garlic and onion, in rats, demonstrating that cycloalliin was absorbed with a relatively low bioavailability: 3.7% at 25 mg/kg and 9.7% at 50 mg/kg [53,101]. They also reported that a deoxidized form of cycloalliin is absorbed better than cycloalliin [53,101]. Nagae et al. examined the pharmacokinetics of SAC, a deoxidized form of alliin, in mice, rats and dogs, revealing that SAC was highly absorbable with oral bioavailabilities ranging between 87% and 102% [11]. Kodera et al. examined the pharmacokinetics of SAC in humans and reported that blood concentrations of SAC lasted long (t_1/2_, >10 h) after the intake of AGE [12]. Recently, Amano et al. investigated the pharmacokinetics, metabolism and excretion of three *S*-alk(en)yl-l-cysteines in AGE, SMC, SAC and S1PC, which are deoxidized forms of methiin, alliin and isoalliin, respectively (Table 2) [28,102]. These three compounds showed good oral absorption in rats and dogs with bioavailabilities of 88–100%. They summarized that the pharmacokinetics of SMC, SAC, and S1PC were characterized by high oral absorption and extensive renal reabsorption, both of which contributed to their long elimination half–lives (5.6–12 h), especially in dogs. In their studies, the pharmacokinetic profiles of S1PC and SAC were shown to be similar in rats and dogs. *N*-Acetylation was the main elimination pathway of SAC and S1PC in both animal species, with the plasma concentration of their *N*-acetylated metabolites being 1/2 to 1/3 of those of the parent forms. Additionally, an in vitro metabolism study revealed that the extent of *N*-acetylated metabolite formation from SAC and S1PC was dependent on the relative activities of *N*-acetylation and deacetylation in the liver and kidney. In rats, SAC and S1PC were primarily eliminated as their *N*-acetylated metabolites into urine, resulting from both renal reabsorption of the unchanged forms and active urinary secretion of the *N*-acetylated metabolites. On the other hand, the *N*-acetylated metabolites of SAC and S1PC were excreted in the urine of dogs to a much smaller extent because dog kidney had extremely high deacetylation activity for the *N*-acetylated metabolites [28,102]. The authors concluded that the kidney plays a critical role in the elimination of SAC and S1PC through renal excretion, reabsorption, and metabolism.

Several studies regarding the safety of AGE and its constituents have been reported [9,12,22,29]. A sub–acute study (one month) of SAC in rats demonstrated its high safety profile with a non-toxic dose of 250 mg/kg (oral dose) [12]. As the use of garlic preparations increased, the possible interactions with prescription medications have become a concern. Macan et al. reported the result of a clinical study that concomitant use of AGE with warfarin, an anticoagulant agent, for 12 weeks did not enhance hemorrhagic events [27]. Amano et al. investigated the effects of SMC, SAC, and S1PC on enzymatic activities of five major isoforms of human cytochrome P450 (CYP): CYP1A2, 2C9, 2C19, 2D6 and 3A4 [29]. Allicin, a hydrophobic compound, which is produced in crashed garlic, but not detected in AGE, inhibited CYP1A2 and CYP3A4 activities with IC_50_ values of 67 and 50–165 μM, respectively. On the contrary, SMC, SAC and S1PC showed no effects on the reactions catalyzed by the human CYP isoforms, even at 1 mM, except in one case: S1PC inhibited a CYP3A4-catalyzed reaction by 31%. These results strongly suggested that these *S*-alk(en)yl-l-cysteines have little potential to cause drug-drug interactions through human CYP inhibition or activation. Gurley et al. reviewed the findings from a number of studies for evaluation of the drug interaction potential of garlic supplements in vitro and in vivo, concluding that commercially available garlic products, including AGE, have a limited potential to produce clinically important herb-drug interactions [103].

## 7. Conclusions

Curiously, since its identification in garlic more than 50 years ago, few reports concerning the biological properties of S1PC have appeared in the medical literature. Recent studies, however, have revealed a host of noteworthy pharmacological effects linked to S1PC. Raw garlic contains only a small amount of S1PC, but its content increases dramatically during the aging process of AGE. S1PC was shown to possess both immunomodulatory and antihypertensive effects in both in vitro and in vivo animal studies. These results are in accordance with previously observed pharmacological effects of AGE in experimental animal and human clinical studies [70,72,88,89]. Furthermore, S1PC showed good pharmacokinetic profiles in rats and dogs with the excellent bioavailability (88–100%), and long elimination half–lives, which may be attributed to its extensive renal reabsorption. Additionally, S1PC had little influence on human CYP activities even at the high concentration of 1 mM, suggesting that S1PC may not cause drug-drug interactions when consumed concomitantly with other medical agents. These results strongly suggest that S1PC is an orally-active component of AGE, which contributes to the prevention and alleviation of lifestyle-related illnesses and immune diseases.

## Figures and Tables

**Table 1:** Altered metabolite levels in plasma after oral administration of S1PC to SHRs. ^a^

Metabolite ^b^	Control vs. WKY ^c^	S1PC vs. Control ^c^
Betaine	↑↑ ^d^	↓
Tryptophan	↓↓	↑↑
LysoPC(16:0)	↓↓	↑↑
LysoPC(18:3)	↓↓	↑↑
LysoPC(20:4)	↓↓	↑
3-(Acetyloxy)-2-hydroxypropyl icosanoate	↑	↓↓
Caproic acid	↑↑	↓

^a^ Table was rewritten by referring to a previous study [21]; ^b^ Blood sample was drawn from the abdominal aorta into vacuum blood collection tubes with EDTA. Samples were analyzed by means of a UltiMate 3000 UHPLC system (Thermo Fisher Scientific, Waltham, MA, USA) coupled with a Q-Exactive quadrupole-Orbitrap mass spectrometer (Thermo Fisher Scientific) [21]; ^c^ Distilled water was orally administrated to WKY rats and SHRs (Control) once a day for 10 weeks. S1PC (6.5 mg/kg) was orally administrated to SHRs once a day for 10 weeks; ^d^ ↑: up-regulated (*p* < 0.05), ↑↑: up-regulated (*p* < 0.01), ↓: down-regulated (*p* < 0.05), ↓↓: down-regulated (*p* < 0.01).

**Table 2:** Pharmacokinetic parameters and excretion levels of SMC, SAC, and S1PC in rats and dogs after oral administration. ^a^

Animal	Compound	C_max_ (mg/L)	T_1/2_ (h)	AUC (mg·h/L)	BA (%)
Rat	SMC ^b^	1.3 ± 0.33	2.6 ± 0.75	6.0 ± 1.7	95.8
	SAC ^c^	3.9 ± 0.64	1.2 ± 0.19	5.1 ± 0.58	91.9
	S1PC ^c^	4.2 ± 0.92	0.56 ± 0.072	3.2 ± 0.34	88.0
Dog	SMC ^b^	2.6 ± 0.62	8.0 ± 1.1	41 ± 5.3	95.5
	SAC ^b^	2.1 ± 0.14	12 ± 1.2	39 ± 3.5	92.0
	S1PC ^b^	2.0 ± 0.22	5.6 ± 0.58	19 ± 2.4	100

^a^ Table was rewritten from [28,102]; ^b^ Oral administration at 2 mg/kg; ^c^ Oral administration at 5 mg/kg.

## Supplementary Files

Supplementary File 1

## References

[1] Koch H.P., Lawson L.D. (1996). The Science and Therapeutic Application of Allium Sativum L and Related Species.

[2] Chauhan N.B. (2005). Multiplicity of garlic health effects and Alzheimer’s disease. J. Nutr. Health Aging.

[3] Ray B., Chauhan N.B., Lahiri D.K. (2011). The “aged garlic extract”: (AGE) and one of its active ingredients *S*-allyl-l-cysteine (SAC) as potential preventive and therapeutic agents for Alzheimer’s disease (AD). Curr. Med. Chem..

[4] Rivlin R. (2001). Historical perspective on the use of garlic. J. Nutr..

[5] Block E. (2010). Garlic and Other Aliums, The Lore and the Science.

[6] Borlinghaus J., Albrecht F., Gruhlke M.C., Nwachukwu I.D., Slusarenko A.J. (2014). Allicin: Chemistry and biological properties. Molecules.

[7] Lawson L.D., Wang Z.J. (1993). Pre-hepatic fate of the organosulfur compounds derived from garlic (*Allim sativum*). Planta Med..

[8] Freeman F., Kodera Y. (1995). Garlic chemistry: Stability of *S*-(2-propyl) 2-propen-1-sulfinothioate (allicin) in blood, solvents, and stimulated physiological fluids. J. Agric. Food Chem..

[9] Amagase H., Petesch B.L., Matsuura H., Kasuga S., Itakura Y. (2001). Intake of garlic and its bioactive components. J. Nutr..

[10] Colin-Gonzalez A.L., Santana R.A., Silva-Islas C.A., Chanez-Cardenas M.E., Santamaria A., Maldonado P.D. (2012). The Antioxidant Mechanisms Underlying the Aged Garlic Extract and *S*-Allylcysteine-Induced Protection. Oxid. Med. Cell Longev..

[11] Nagae S., Ushijima M., Hatono S., Imai J., Kasuga S., Matsuura H., Itakura Y., Higashi Y. (1994). Pharmacokinetics of the garlic compound *S*-allylcysteine. Planta Med..

[12] Kodera Y., Suzuki A., Imada O., Kasuga S., Sumioka I., Kanezawa A., Taru N., Fujikawa M., Nagae S., Masamoto K. (2002). Physical, chemical, and biological properties of *S*-allylcysteine, an amino acid derived from garlic. J. Agric. Food. Chem..

[13] Sugii M., Suzuki T., Nagasawa S. (1963). Isolation of (–)*S*-propenyl-l-cysteiene from garlic. Chem. Abstr..

[14] Carson J.F., Boggs L.E. (1966). The synthesis and base-catalyzed cyslization of (+)– and (–)-*cis*-*S*-(1-propenyl)-l-cysteine sulfoxides. J. Org. Chem..

[15] Nishimura H., Mizuguchi A., Mizutani J. (1975). Stereoselective synthesis of *S*-(*trans*-prop-1-enyl)-cysteine sulphoxide. Tet. Lett..

[16] Namyslo J.C., Stanitzek C. (2006). A palladium–catalyzed synthesis of isoalliin, the main cysteine sulfoxide in onion (*Allim cepa*). Synthesis.

[17] Lee A., Kim J.N., Choung D.H., Lee H.K. (2011). Facile synthesis of *trans*-*S*-1-propenyl-L-cysteine sulfoxide (Isoalliin) in onions (*Allium cepa*). Bull. Korean Chem. Soc..

[18] Kodera Y., Matsutomo T., Itoh K. (2015). The evidence for the production mechanism of *cis*-*S*-1-propenylcysteine in aged garlic extract based on a model reaction approach using its isomers and deuterated solvents. Planta Med. Lett..

[19] Matsutomo T., Kodera Y. (2016). Development of an analytical method for sulfur compounds in aged garlic extract with the use of a postcolumn high performance liquid chromatography method with sulfur-specific detection. J. Nutr..

[20] Suzuki J., Yamaguchi T., Natsutomo T., Amano H., Morihara N., Kodera Y. (2016). *S*-1-Propenylcysteine promotes the differentiation of B cell into IgA-producing cells by the induction of Erk1/2-dependent *Xbp1* expression in Peyer’s patches. Nutrition.

[21] Matsutomo T., Ushijima M., Kodera Y., Nakamoto M., Takashima M., Morihara N., Tamura K. (2017). Metabolomic study on the antihypertensive effect of *S*-1-propenylcysteine in spontaneously hypertensive rats using liquid chromatography coupled with quadrupole-Orbitrap mass spectrometry. J. Chromatogr. B.

[22] Harauma A., Moriguchi T. (2006). Aged garlic extract improves blood pressure in spontaneous hypertensive rats more safely than raw garlic. J. Nutr..

[23] Sridharan K., Sivaramakrishnan G. (2016). Interaction of Citrus Juices with Cyclosporine: Systematic Review and Meta-Analysis. Eur. J. Drug. Metab. Pharmacokinet..

[24] Gurley B.J., Fifer E.K., Gardner Z., Wienkers L. (2012). Phytochemicals modulators of human drug metabolism: Drug interactions with fruits, vegetables and botanical dietary supplements. Encyclopedia of Drug Metabolism and Interactions.

[25] Tapaninen T., Neuvonen P.J., Niemi M. (2010). Grapefruit juice greatly reduces the plasma concentrations of the OATP2B1 and CYP3A4 substrate aliskiren. Clin. Pharmacol. Ther..

[26] Vaes L.P., Chyka PA. (2000). Interactions of warfarin with garlic, ginger, ginkgo, or ginseng: Nature of the evidence. Ann. Pharmacother..

[27] Macan H., Uykimpang R., Alconcel M., Takasu J., Razon R., Amagase H., Niihara Y. (2006). Aged garlic extract may be safe for patients on warfarin therapy. J. Nutr..

[28] Amano H., Kazamori D., Itoh K. (2016). Pharmacokinetics and *N*-acetylation metabolism of *S*-methyl-l-cysteine and *trans*-*S*-1-propenyl-l-cysteine in rats and dogs. Xenobiotica.

[29] Amano H., Kazamori D., Itoh K. (2016). Evaluation of the effects of *S*-allyl-l-cysteine, *S*-methyl-l-cysteine, *trans*-*S*-1-propenyl-l-cysteine, and their *N*-acetylated and *S*-oxidized metabolites on human CYP activities. Biol. Pharm. Bull..

[30] Block E. (1985). The chemistry of garlic and onions. Sci. Am..

[31] Block E. (1992). The organosulfur chemistry of the genus *Allium*—Implication for the organic chemistry of sulfur. Angew. Chem. Int. Ed. Engl..

[32] Lawson L.D., Wang Z.Y., Hughes B.G. (1991). γ-Glutamyl-*S*-alklycysteines in garlic and other *Allium* spp.: Precursor of age-dependent *trans*-1-propenyl thiosulfinates. J. Nat. Prod..

[33] Masters P.M., Friedman M. (1979). Racemization of amino acids in alkali-treated food proteins. J. Agric. Food Chem..

[34] Liardon R., Hurrell R.F. (1983). Amino acid racemization in heated and alkali-treated proteins. J. Agric. Food Chem..

[35] Smith G.G., Sivakua T. (1983). Mechanism of the racemization of amino acids. Kinetics of racemization of arylglycines. J. Org. Chem..

[36] Turnbull A., Galpin I.J., Collin H.A. (1980). Comparison of the onion plant (*Allium cepa*) and onion tissue culture. III. Feeding of ^14^C lS1PC has two forms of isomersabeled precursor of the flavor precursor compounds. New Phytol..

[37] Lancaster J.E., Show M.L. (1989). γ-Glutamyl peptides in the biosynthesis of *S*-alk(en)yl-l-cysteine sulphoxides (flavor precursors) in *Allium*. Phytochemistry.

[38] Yoshimoto N., Onuma M., Mizuno S., Sugino Y., Nakabayashi R., Imai S., Tsuneyoshi T., Sumi S., Saito K. (2015). Identification of a flavine-containing *S*-oxygenating monooxygenase involved in alliin biosynthesis in garlic. Plant J..

[39] Yoshimoto N., Yabe A., Sugino Y., Murakami S., Sai-Ngam N., Sumi S., Tsuneyoshi T., Saito K. (2015). Garlic γ-glutamyl transpeptidases that catalyze deglutamylation of biosynthetic intermediate of alliin. Front. Plant Sci..

[40] Parry R.J., Sood G.R. (1989). Investigations of the biosynthesis of *trans*-(+)-*S*-1-propenyl-l-cysteine sulfoxide in onions (*Allium cepa*). J. Am. Chem. Soc..

[41] Parry R.J., Lii F.L. (1991). Investigations of the biosynthesis of *trans*-(+)-*S*-1-propenyl-l-cysteine sulfoxide. Elucidation of the stereochemistry of the oxidative decarboxylation process. J. Am. Chem. Soc..

[42] Schlaich N.L. (2007). Flavin-containing monooxygenase in plants: Looking beyond detox. Trends Plant Sci..

[43] Carson J.F., Wong F.P. (1961). The volatile flavor components of onion. J. Agric. Food Chem..

[44] Brondnitz M.H., Pollck C.L., Vallon P.P. (1969). Flavor components of onion oil. J. Agric. Food Chem..

[45] Cai X.L., Uden P.C., Block E., Zhang X., Quimby B.D., Sullivan J.J. (1994). Allium chemistry: Identification of natural abundance organoselenium volatiles from garlic, elephant garlic, onion, and Chinese chive using headspace gas chromatography with atomic emission detection. J. Agric. Food Chem..

[46] Itakura Y., Ichikawa M., Mori Y., Okino R., Udayama M., Morita T. (2001). How to distinguish garlic from the other *Allium* vegetables. J. Nutr..

[47] Brodnitz M.H., Pascale J.V., Derslice L.V. (1971). Flavor components of garlic extract. J. Agric. Food Chem..

[48] Block E. (1993). Flavor artifacts. J. Agric. Food Chem..

[49] Block E., Calvey E.M., Mussinan C.L., Keelan M.E. (1994). Facts and artifacts in Allium chemistry Sulfur Compounds in Foods. ACS Symposium Series 564.

[50] Lawson L.D., Wang Z.J., Hughes B.G. (1991). Identification and HPLC quantitation of the sulfides and dialk(en)yl thiosulfides in commercial garlic products. Planta Med..

[51] Yamasaki Y., Tokunaga T., Okuno T. (2005). Quantitative determination of eleven flavor precursor (*S*-alke(en)yl cysteine derivatives) in the garlic with an HPLC method. Nippon Shoyaku Kagaku Kaishi.

[52] Thomas D.J., Parkin K.L. (1994). Quantification of alk(en)yl-l-cysteine sulfoxides and related amino acids in Alliums by high-performance liquid chromatography. J. Agric. Food Chem..

[53] Ichikawa M., Ide N., Yoshida J., Yamaguchi H., Ono K. (2006). Determination of seven organosulfur compounds in garlic by high-performance liquid chromatography. J. Agric Food Chem..

[54] Kubec R., Dadakova E. (2009). Chromatographic methods for determination of *S*-substituted cysteine—A comparative study. J. Chromatogr. A.

[55] Yu T.H., Wu C.M., Rosen R.T., Hartman T.G., Ho C.T. (1994). Volatile compounds generated from thermal degradation of alliin and deoxyalliin in an aqueous solution. J. Agric. Food Chem..

[56] Kubec R., Svobodova M., Velisek J. (1999). Gas chromatographic determination of *S*-alk(en)ylcysteine sulfoxides. J. Chromatogr. A.

[57] Kubec R., Dadakova E. (2008). Quantitative determination of *S*-alk(en)ylcysteine-*S*-oxides by micellar electrokinetic capillary chromatography. J. Chromatogr. A.

[58] Awwad H.K., Adelstein S.J. (1966). A quantitative method for the determination of the specific radioactivity of sulfur-containing amino acids separated by paper chromatography. Anal. Biochem..

[59] Fowler B., Robins A.J. (1972). Methods for the quantitative analysis of sulphur–containing compounds in physiological fluids. J. Chromatogr..

[60] Kawano S., Yasui Y., Hayashi M., Shono T., Chone Y., Hirai-(Emoto) S., Aoki M. (1997). Post-column derivatization using hexaiodoplatinate for determination of sulfur-containing amino acids by high performance liquid chromatography. Shimadzu Hyoron.

[61] Dirsch V.M., Kiemer A.K., Wagner H., Vollmar A.M. (1998). Effect of allicin and ajoene, two compounds of garlic, on inducible nitric oxide synthase. Atherosclerosis.

[62] Kang N.S., Moon E.Y., Cho C.G., Pyo S. (2001). Immunomodulating effect of garlic component, allicin, on murine peritoneal macrophages. Nutr. Res..

[63] Feng Y., Zhu X., Wang Q., Jiang Y., Shang H., Cui L., Cao Y. (2012). Allicin enhances host pro–inflammatory immune responses and protects against acute murine malaria infection. Malar. J..

[64] Chang H.P., Chen Y.H. (2005). Differential effects of organosulfur compounds from garlic oil on nitric oxide and prostaglandin E2 in stimulated macrophages. Nutrition.

[65] Chang H.P., Hung S.Y., Chen Y.H. (2005). Modulation of cytokine secretion by garlic oil derivatives is associated with suppressed nitric oxide production in stimulated macrophages. J. Agric. Food Chem..

[66] Liu K.L., Chen H.W., Wang R.Y., Lei Y.P., Sheen L.Y., Lii C.K. (2006). DATS reduces LPS-induced iNOS expression, NO production, oxidative stress, and NF-kappaB activation in RAW264.7 macrophages. J. Agric. Food Chem..

[67] Quintero-Fabian S., Ortuno-sahagun D., Vazquez-Carrera M., Lopez-Roa R.I. (2013). Alliin, a garlic (*Allium sativum*) compound, prevents LPS-induced inflammation in 3T3-L1 adipocytes. Mediat. Inflamm..

[68] Salman H., Bergman M., Bessler H., Punsky I., Djaldetti M. (1999). Effect of a garlic derivative (Alliin) on peripheral bloodcell immune responses. Int. J. Immunopharm..

[69] Kyo E., Uda N., Kasuga S., Itakura Y. (2001). Immunomodulatory effects of aged garlic extract. J. Nutr..

[70] Ishikawa H., Saeki T., Otani T., Suzuki T., Shimozuma K., Nishino H., Fukuda S., Morimoto K. (2006). Aged garlic extract prevents a decline of NK number and activity in patients with advanced cancer. J. Nutr..

[71] Fallah-Rostami F., Tabari M.A., Esfandiari B., Aghajanzadeh H., Behzadi M.T. (2013). Immunomodulatory activity of aged garlic extract against implanted fibrosarcoma tumor in mice. N. Am. J. Med. Sci..

[72] Nantz M.P., Rowe C.A., Muller C.E., Creasy R.A., Stanilka J.M., Percival S.S. (2012). Supplementation with aged garlic extract improves both NK and γδ–T cell function and reduces the severity of cold and flu symptoms: A randomized, double-blind, placebo-controlled nutrition intervention. Clin. Nutr..

[73] Fagarasan S., Honjo T. (2003). Intestinal IgA synthesis: Regulation of front-line body defences. Nat. Rev. Immunol..

[74] Macpherson A.J., Geuking M.B., McCoy K.D. (2012). Homeland security: IgA immunity at the frontiers of the body. Trends Immunol..

[75] Macpherson A.J., McCoy K.D., Johansen F.E., Brandtzaeg P. (2008). The immune geography of IgA induction and function. Mucosal Immunol..

[76] Tsuji M., Suzuki K., Kinoshita K., Fagarasan S. (2008). Dynamic interactions between bacteria and immune cells leading to intestinal IgA synthesis. Semin. Immunol..

[77] Swann J.B., Hayakawa Y., Zerafa N., Sheehan K.C., Scott B., Schreiber R.D., Hertzog P., Smyth M.J. (2007). Type I IFN contributes to NK cell homeostasis, activation, and antitumor function. J. Immunol..

[78] Guan J., Miah S.M., Wilson Z.S., Erick T.K., Banh C., Brossay L. (2014). Role of type I interferon receptor signaling on NK cell development and functions. PLoS ONE.

[79] Madera S., Rapp M., Firth M.A., Beilke J.N., Lanier L.L., Sun J.C. (2016). Type I IFN promotes NK cell expansion during viral infection by protecting NK cells against fratricide. J. Exp. Med..

[80] Iwakoshi N.N., Pypaert M., Glimcher L.H. (2007). The transcription factor XBP-1 is essential for the development and survival of dendritic cells. J. Exp. Med..

[81] Martinon F., Chen X., Lee A.H., Glimcher L.H. (2010). TLR activation of the transcription factor XBP1 regulates innate immune responses in macrophages. Nat. Immunol..

[82] Mujaj S., Gandhi M., Vari F., Nourse J. (2012). Modulation of the unfolded protein response via XBP1 splicing: A novel mechanism that regulates Natural Killer cell effector function (172.10). J. Immunol..

[83] Zheng X., Wang Y., Wei H., Sun R., Tian Z. (2009). LFA-1 and CD2 synergize for the erk1/2 activation in the natural killer (NK) cell immunological synapse. J. Biol. Chem..

[84] Global Health Observatory (GHO) Data: Raised Blood Pressure. http://www.who.int/gho/ncd/risk_factors/blood_pressure_prevalence_text/en/.

[85] Sim J.J., Bhandari S.K., Shi J., Reynolds K., Calhoun D.A., Kalantar-Zadeh K., Jacobsen S.J. (2015). Comparative risk of renal, cardiovascular, and mortality outcomes in controlled, uncontrolled resistant, and nonresistant hypertension. Kidney Int..

[86] Patel P., Ordunez P., DiPette D., Escobar M.C., Hassell T., Wyss F., Hennis A., Asma S., Angell S. (2016). Improved blood pressure control to reduce cardiovascular disease morbidity and mortality: The standardized hypertension treatment and prevention project. J. Clin. Hypertens..

[87] Ried K. (2016). Garlic Lowers Blood Pressure in Hypertensive Individuals, Regulates Serum Cholesterol, and Stimulates Immunity: An Updated Meta-analysis and Review. J. Nutr..

[88] Ried K., Frank O.R., Stocks N.P. (2013). Aged garlic extract reduces blood pressure in hypertensives: A dose-response trial. Eur. J. Clin. Nutr..

[89] Ried K., Frank O.R., Stocks N.P. (2010). Aged garlic extract lowers blood pressure in patients with treated but uncontrolled hypertension: A randomised controlled trial. Maturitas.

[90] Okamoto K., Aoki K. (1963). Development of a strain of spontaneously hypertensive rats. Jpn. Circ. J..

[91] Fukuda S., Tsuchikura S., Iida H. (2004). Age-related changes in blood pressure, hematological values, concentrations of serum biochemical constituents and weights of organs in the SHR/Izm, SHRSP/Izm and WKY/Izm. Exp. Anim..

[92] Guo Z., Muller D., Pentz R., Kress G., Siegers C.-P. (1990). Bioavailability of sulphur-containing ingredients of garlic in the rat. Planta Med..

[93] Lachmann G., Lorenz D., Radeck W., Steiper M. (1994). The pharmacokinetics of the S35 labeled labeled garlic constituents alliin, allicin and vinyldithiine. Arzneimittelforschung.

[94] Cruz C., Correa-Rotter R., Sánchez-González D.J., Hernández-Pando R., Maldonado P.D., Martínez-Martínez C.M., Medina-Campos O.N., Tapia E., Aguilar D., Chirino Y.I. (2007). Renoprotective and antihypertensive effects of S-allylcysteine in 5/6 nephrectomized rats. Am. J. Physiol. Renal Physiol..

[95] Lever M., Atkinson W., George P.M., Chambers S.T. (2007). Sex differences in the control of plasma concentrations and urinary excretion of glycine betaine in patients attending a lipid disorders clinic. Clin. Biochem..

[96] Wang Y., Liu H., McKenzie G., Witting P.K., Stasch J.P., Hahn M., Changsirivathanathamrong D., Wu B.J., Ball H.J., Thomas S.R. (2010). Kynurenine is an endothelium-derived relaxing factor produced during inflammation. Nat. Med..

[97] Hirayama T., Ogawa Y., Tobise K., Kikuchi K. (1998). Mechanism of endothelium-dependent vasorelaxation evoked by lysophosphatidylcholine. Hypertens. Res..

[98] Pushpendran C.K., Devasagayam T.P., Chintalwar G.J., Banerji A., Eapen J. (1980). The metabolic fate of (35*S*)-diallyl disulphide in mice. Experientia.

[99] Devasagayam T.P., Pushpendran C.K., Eapen J. (1982). Diallyl disulphide induced changes in microsomal enzymes of suckling rats. Indian J. Exp. Biol..

[100] Wang W., Tang J., Peng A. (1989). The isolation, identification, and bioactivities of selenoproteine in selsenium-rich garlic. Shengwu Huaxue Zazhi.

[101] Matsutomo T., Ichikawa M., Kodera Y. (2008). Pharmoacokinetics of water soluble organosulfur compounds from garlic. J. Clin. Biochem. Nutr..

[102] Amano H., Kazamori D., Itoh K., Kodera Y. (2015). Metabolism, excretion, and pharmacokinetics of *S*-allyl-l-cysteine in rats and dogs. Drug Metab. Dispos..

[103] Gurley B.J., Fifer K.E., Gardner Z. (2012). Pharmacokinetic herb–drug interactions (Part 2): Drug interactions involving popular botanical dietary supplements and their clinical relevance. Planta Med..

