# Supplementary material for: Chemical and Biological Properties of S-1-Propenyl-l-Cysteine in Aged Garlic Extract
Source: Molecules. 2017 Mar 31;22(4):570. doi: 10.3390/molecules22040570 (PMC6154623; doi:10.3390/molecules22040570)
Supplement: Supplementary File 1 [file molecules-22-00570-s001.pdf]

Point to Point Reply for MS ID#: molecules-180259

First of all, we greatly appreciate two reviewers for their kind guidance and comments on our manuscript.

We have taken all the comments and suggestions into account in the revised version as described below. Please see the yellow highlighted words/sentences for the changes made.

****

Reviewer 1 COMMENTS

General comment:

The topic of the review article is of interest to me. Its contents are of scientific relevance. However, the article does not read well. The authors tend to be repetitive in their writing. The authors also need to improve on the sentence structure and grammar. In some instances, I would need to refer to the cited reference to understand the written sentence.

I would recommend the article to be sent for professional English editing.

Reply:

We deeply appreciate the comment. Your comment was very helpful for our revision. We have revised our manuscript according to other reviewer comments and correction by professional English editing.

We thought that several "the repetitive writing" relating to stability/instability, bioavailability, and comparison with the properties of other compound, were necessary to explain the properties of S1PC. However, we have changed the several parts as "the repetitive writing."

Lines 24-27 in abstract and lines 76-79 in introduction have been changed as "the repetitive writing." Please see new lines 24-25 and 72-75.

We have omitted "a stereoisomer of S1PC" in line 147. Please see new line 144.

We have omitted "a precursor of S1PC" in line 245. Please see new line 244.

We have omitted "S1PC has two isomers, *cis*– and *trans*–S1PC [13–18]" in line 261. Please see new line 259.

We have rewritten “beginning of 6. Biological Properties of S-1-propenyl-L-Cysteine” in lines 281-288. Please see new lines 278-286.

****

Reviewer 2 COMMENTS

General Comment:
The manuscript is prepared very precisely. The theme is very actual because of unhealthy life style and civilization diseases occurrence of current human population as well as necessity of bioactive compounds intake in food.

Reply: We appreciate the comment above. Your comment was very helpful for our revision. We have revised our manuscript according to other reviewer comments and correction by professional English editing.

Reviewer Comments:
Line 28 - replace Latin name (*Allium sativum*) for (*Allium sativum* L.),
Reply:
We have reworded the word “*Allium sativum* ” to the word “*Allium sativum* L.”. Please see the new line 26.

Reviewer Comments:
Line 83 - replace Latin name (*Allium cepa*) for (*Allium cepa* L.),
Reply:
We have reworded the word “*Allium cepa*” to the word “*Allium cepa* L.”. Please see the new line79.

Reviewer Comments:
Line 106 **Namysle and Stanizek** – in the text **Namyslo and Stanitzek** – in References,
Reply:
We have chacked and reworded name “Namysle and Stanizek” in text to “Namyslo *et al.*” in text. Please see the new lines 103-104 .

Reviewer Comments:
Line 108 - more correct citation **Lee et al.** and not **Lee and Kim** - because in the

bibliography lists more authors,

Reply:

We have reworded the citation “Lee and Kim” to “Lee *et al.*” Please see the new line 107.

Reviewer Comments:

Line 108 - Authors should check the References and compare it with the text of the manuscript, as some sources are missing (e.g. line 108 **Ortar et al.(1995)** is missing in References,

Reply:

We have ommited “based on the report by Ortar *et al.* in 1995”. Although the report by Ortar *et al.* is an advance report about the palldium-catalyzed caupling, Namyslo *et al.* explained it enough. Please see the new lines 104-05.

Reviewer Comments:

Line 159 - I propose to shorten the name of figure and give a description of figure directly into the text (for example **Figure 4**),

Reply:

We thought the name of “**Figure 4.** Biosynthesis of sulfur–containing compounds in garlic ”was short name to explain the content of figure. We did not change it. Please see the new line 156.

Reviewer Comments:

Line 169 - Check citation **Yoshimoto et al. [39,39],**

Reply:

We checked and changed the citation“ “[39, 39]” to “[38, 39]”. Please see the new line 166.

Reviewer Comments:

Line 295 - Correct vinyldthiins for vinyldithiins

Reply:

We have reworded the word “vinyldthiins ” to the word “vinyldithiins”. Please see the new line 293.

****

Reviewer 3　COMMENTS

General Comments:

The review was interesting and should be of value to researchers in the area of botanical dietary supplements, especially those with a focus on garlic and its sulfur-containing phytochemicals. The major issue I had with the manuscript was the need for significant editing with regard to English grammar and syntax. As a favor to the authors, I have made a large number of editorial suggestions. In the future the authors should have someone proficient in the English language proof read their manuscripts before submitting them to Molecules.

Reply:

We deeply appreciate the comment and huge number of English editing. Your comment was very helpful for our revision. We have revised our manuscript according to other reviewer comments and correction by professional English editing.

Reviewer comment:

Line 18. Remove "the" before "Immunomodulatory."

Reply:

We have removed the word "the" before "Immunomodulatory." Please see the new line 18.

Reviewer comment:

Line 20. Add "A" to the beginning of this sentence.

Reply:

We have added the word "A" to the beginning of sentence in the line 20. Please see the new line 19.

Reviewer comment:

Line 34. Insert "a" after "have," and replace "by the" with "in." Make "human" plural.

Reply:

Finally, we have rewritten this sentence to "Several plants belonging to the genus *Allium* have been historically used by humans" according to correction of professional deiting. Please see the new line 32.

Reviewer comment:

Line 38. Remove "the" before "beneficial." Remove "their."

Reply:

We have removed the word "the" before "beneficial" and "their." Finally, we have rewritten “the lines 37-40” to ”Many people in the world still use garlic and its preparations for health benefits, as several scientific studies have reported its following properties; antioxidative, antithrombotic, hypolipidemic, hypoglycemic, antihypertensive, and anti–Alzheimer’s disease effects [2, 3].” Please see the new lines 35-37.

Reviewer comment:
Line 41. Remove the phrase "through their experiences even." Make "evidences" singular.
Reply:
We have removed the phrase "through their experiences even." We have made "evidences" singular. Please see the new line 38.

Reviewer comment:
Line 43. I seriously doubt that the discovery of the alliin-allinase was "epoch-making." Please use another adjective here.
Reply:
We have replaced the words "epoch-making" with “noteworthy.” Please see the new line 40.

Reviewer comment:
Line 44. Change "had" to "has."
Reply:
We have changed the word "had" to "have" according to the correction by the professional English editing. Please see the new line 41.

Reviewer comment:
Line 45. Insert "have" after "scientists."
Reply:
We have inserted the word "have" after "scientists." Please see the new line 42.

Reviewer comment:
Line 47. Insert "many" before "preparations."
Reply:
We have inserted the word "many" before "preparations." Please see the new line 44.

Reviewer comment:

Line 49. Replace "after" with "since."

Reply:

We have replaced the word "after" with "since." Please see the new line 46.

Reviewer comment:

Line 51. Add commas after "allicin" and "instability."

Reply:

We have added commas after "allicin" and "instability." Please see the new line 48.

Reviewer comment:

Line 57. Delete "by many scientists."

Reply:

We have deleted the words "by many scientists." We have rewritten “the line 57-58” to “SAC has been extensively studied and shown to possess various biological activities, such as anticancer, antioxidant, cholesterol–lowering, anti–hepatotoxic, and neuroprotective effects” according to the correction by the professional English editing. Please see the new line 54-55.

Reviewer comment:

Line 64. Replace "was" with "were."

Reply:

We have replaced the word "was" with "were." Please see the new line 61.

Reviewer comment:

Line 65. Make "report" plural. Change "suggested" to "suggest."

Reply:

We have made "report" plural and changed the word "suggested" to "suggest." Please see the new lines 61 and 62 .

Reviewer comment:

Line 67. Make "preparations" plural. Replace "to be useful for" with "regarding."

Reply:

We have made "preparations" plural. We have replaced the words "to be useful for" with "in" according to the correction by the professional English editing. Please see the new lines 63.

Reviewer comment:

Line 70. Several of these references refer to grapefruit juice only. I would suggest replacing references 23-25 with the following reference which includes not only a review of grapefruit juice-mediated interactions, but a host of other clinically relevant herb-drug interactions: Gurley BJ, Fifer EK, Gardner Z. “Phytochemical Modulators of Human Drug Metabolism: Drug Interactions with Fruits, Vegetables and Botanical Dietary Supplements” in Encyclopedia of Drug Metabolism and Interactions, L. Wienkers, ed., John Wiley & Sons, Inc. Hoboken, NJ (published online May15, 2012).

Reply:

We have replaced the “reference [24]” with “reviewer recommended one: Gurley BJ, Fifer EK, Gardner Z. “Phytochemical Modulators of Human Drug Metabolism: Drug Interactions with Fruits, Vegetables and Botanical Dietary Supplements” in Encyclopedia of Drug Metabolism and Interactions, L. Wienkers, ed., John Wiley & Sons, Inc. Hoboken, NJ (published online May15, 2012).” Please see the new line 67, and lines 572-575 in the citation section.

Reviewer comment:

Line 77. Replace the second "the" with "its." Remove the third and fourth "the."

Reply:

We have replaced the second "the" with "its" in the line 77. We have removed the third and fourth "the" in the line 77. Please see the line 73.

Reviewer comment:

Line 78. Remove "the" in both instances in this line.

Reply:

We have removed the word "the" in both instances in line 78. Please see the new line 74.

Reviewer comment:

Line 79. Remove "the" in this line.

Reply:

We have removed the word "the" in the line 79. Please see the new line 75.

Reviewer comment:

Line 83. Insert "as" before "trans-S1PCSO."

Reply:

We have inserted the word "as" before "trans-S1PCSO." Please see the new line 79.

Reviewer comment:
Line 85. The phrase "means of purification steps" makes no sense here. Not sure what was intended with this phrase. Please revise.
Lines 86. Not sure what you are intending to infer with the phrase "its organic synthesis method has been made." Please revise this entire sentence as it makes no sense in its current form.
Reply:
We have revised the line 84–87 and rewritten as ", a biologically active compound that is beneficial for human health [5] (pp. 60–99, pp. 107–132, pp. 282–297), [16, 17, 30, 31]. The content of this compound in onion is less than 0.2% [16], and its purification is difficult because many compounds with similar chemical characteristics exist in onion. Therefore, its organic synthesis method has been investigated to reveal sulfur chemistry in onion and other *Allium* vegetables, including the preparation of S1PC as an intermediate of S1PCSO [14–17]" according to the suggestion of reviewer and the correction by the professional English editing. Please see the new lines 80-85.

Reviewer comment:
Line 90. Insert "have" after "researchers."
Reply:
We have inserted the word "have" after "researchers." Please see the new line 88.

Reviewer comment:
Line 97. Remove "form." Replace "the" with "an." Replace "and" with "with the."
Reply:
We have removed the word "form." We have rewritten "This method gave both cis- and trans-fprm S1PC in the almost equal ration, and cis-form" to "This method yieled both *cis*– and *trans*–S1PC in almost equal amounts, and the *cis*–form" according to the correction by professional English editing. Please see the new line 95.

Reviewer comment:
Line 98. Remove "crystalline was."
Reply:
We have removed the words "crystalline" and rewritten "crystalline was mainly" to "was mainly." Please see the new lines 95-96.

Reviewer comment:

Line 99. Replace "handle" with "perform."

Reply:

We have replaced the word "handle" with "perform." Please see the new line 97.

Reviewer comment:

Line 100. Replace "form compound in high grade of" with "isomer with high."

Reply:

We have replaced the words "form compound in high grade of" with "isomer with high." Please see the new line 98.

Reviewer comment:

Line 101. Insert "the" after "that."

Reply:

We have inserted the word "the" after "that." Please see the new line 98.

Reviewer comment:

Line 102. Replace "total 5 steps through the," with "a 5-step process involving."

Reply:

We have replaced the words "total 5 steps through the," with "a 5-step process involving." Please see the new line 100.

Reviewer comment:

Line 106. Insert "a" before "sample."

Reply:

We did not use the word "sample" in the line 105 – 108. Therefore, we have not inserted the word "a" anywhere. Please see the new line corresponding to the previous lines 103-104.

Reviewer comment:

Line 108. Remove "step."

Reply:

We have removed the word "step." Please see the new line 106.

Reviewer comment:

Line 112. Remove "the."

Reply:

We have removed the word "the." Please see the new line 109.

Reviewer comment:

Line 113. Remove "form."

Reply:

We have removed the word "form." Please see the new line 110.

Reviewer comment:

Line 125. Insert "biosynthetically" after "compounds."

Reply:

We have inserted the word "biosynthetically" after "compounds." Please see the new line 122.

Reviewer comment:

Line 126. Remove "via their biosynthesis."

Reply:

We have removed the words "via their biosynthesis." Please see the new line 123.

Reviewer comment:

Line 131. Remove "the."

Reply:

We have removed the word "the." Please see the new line 128.

Reviewer comment:

Line 132. Remove "the."

Reply:

We have removed the word "the." Please see the new line 129.

Reviewer comment:

Line 134. Remove "genus."

Reply:

We have removed the word "genus." Please see the new line 131.

Reviewer comment:

Line 136. Remove "the."

Reply:

We have removed the word "the." Please see the new line 133.

Reviewer comment:

Line 137. Insert "In onion," before "after." Add "the" before "β-carboxypropenyl."

Reply:

We have inserted the words "In onion," before "after" and added the word "the" before "β-carboxypropenyl." Please see the new line 133-134.

Reviewer comment:

Line 138. Insert "to" before "yield."

Reply:

We have inserted the word "to" before "yield." Please see the new line 135.

Reviewer comment:

Line 140. Remove "reaction in onion."

Reply:

We have removed the words "reaction in onion." Please see the new line 137.

Reviewer comment:

Line 148. Insert "the" before "allyl."

Reply:

We have inserted the word "the" before "allyl." Please see the new line 145.

Reviewer comment:

Line 150. Replace "was" with "is."

Reply:

We have replace the word "was" with "is." Please see the new line 146.

Reviewer comment:

Line 159. Replace "Alliin" with "The alliin."

Reply:

We have replaced the word "Alliin" with "The alliin." We have also inserted the compound number "(6)" after the word "alliin." Please see the new line 156.

Reviewer comment:

Line 161. Remove "pathway."

Reply:

We have removed the words "pathway." Please see the new line 158.

Reviewer comment:

Line 163. Replace "was not still" with "remains to be"

Reply:

We have replaced the words "was not still" with "remains to be" Please see the new line 161.

Reviewer comment:

Line 164. Replace "S-oxygenete" with "S-oxygenates."

Reply:

We have replaced the word "S-oxygenete" with "S-oxygenates." Please see the new line 161.

Reviewer comment:

Line 166. Replace "was drawn" with "is."

Reply:

We have replaced the word "was drawn" with "is." Please see the new line 164.

Reviewer comment:

Line 167. Replace "literature" with "published mechanisms."

Reply:

We have replace the word "literature" with "published mechanisms." We have also rewritten “Illustration was drawn based on literature” to “Illustration is based on mechanisms published in previous reports.” Please see the new line 164.

Reviewer comment:

Line 168. Remove "in this figure." Make "reaction" plural.

Reply:

We have removed the words "in this figure" and made "reaction" plural. Please see the new lines 165 and 166.

Reviewer comment:

Line 172. Insert "the" before "amount."
Reply:
We have inserted the word "the" before "amount." Please see the new line 170.

Reviewer comment:
Line 175. Insert a comma after "weight." Replace "that" with "which."
Reply:
We have inserted a comma after "weight" and replaced the word "that" with "which." Please see the new line 173.

Reviewer comment:
Line 176. Insert "as a sulfur storage molecule in raw garlic" after "abundant." Replace the comma at the end of the line with a colon.
Reply:
We have inserted the words "as a sulfur storage molecule in raw garlic" after "abundant." We have replaced the comma at the end of the line 176 with a colon. Please see the new lines 174 and 175.

Reviewer comment:
Line 177. Remove "reaction" at both instances in this line.
Reply:
We have removed the "reaction" at both instances in this line. Please see the new line 175.

Reviewer comment:
Line 181. Replace "undergoes" with "occurs." Insert "the" after "during."
Reply:
We have replaced the word "undergoes" with "occurs" and inserted the word "the" after "during." Please see the new line 179.

Reviewer comment:
Line 182. Replace "reaction order" with "synthetic."
Reply:
We have replaced the words "reaction order" with "synthetic." Please see the new line 180.

Reviewer comment:
Line 183. Remove "of synthesis."
Reply:
We have removed the words "of synthesis." Please see the new line 180.

Reviewer comment:
Line 189. Replace "into the" with "as." Remove "ones."
Reply:
We have replaced the words "into the" with "as" and removed the word "ones." Please see the new line 186.

Reviewer comment:
Line 190. Make "method" plural. Replace "has" with "have." Replace "is" with "are."
Reply:
We have made "method" plural. We have replaced the word "has" with "have" and the word "is" with "are." We have rewritten "chromatographic (GC) methods has an outstanding separation ability and is" to "chromatographic (GC) methods have outstanding separation abilities and are" according to correction by the professional English editing. Please see the new line 187.

Reviewer comment:
Line 191. Remove the first "the."
Reply:
We have removed the first "the." Please see the new line 188.

Reviewer comment:
Line 192. Replace "were a lot of" with "are numerous."
Reply:
We have replaced the words "were a lot of" with "are numerous." Please see the new line 189.

Reviewer comment:
Line 193. Replace "In the" with "During." Remove "we have to consider the."
Reply:
We have replaced the words "In the" with "During" and removed the words "we have to consider the." Please see the new line 190.

Reviewer comment:
Line 194.　Insert "must be considered," after "measurement."
Reply:
We have inserted the words "must be considered," after "measurement." Please see the new line 191.

Reviewer comment:
Line 195.　Replace "a separation area, a" with "the."
Reply:
We have replaced the words "a separation area, a" with "in the" according to correction by the professional English editing. Please see the new line 192.

Reviewer comment:
Line 196.　Make "equipments" singular.
Reply:
We have made "equipments" singular. Please see the new line 192.

Reviewer comment:
Line 197.　Remove "widely" after "used" and place it after "been."
Reply:
We have removed the word "widely" after "used" and placed it after "been." Please see the new line 194.

Reviewer comment:
Line 204.　Replace "the" with "an."
Reply:
We have replaced the word "the" with "an." Please see new line 201.

Reviewer comment:
Line 205.　Replace "interfered" with "partially obstructed."　Remove "that."
Reply:
We have replaced the word "interfered" with "partially obstructed" and removed the word "that." Please see the new line 202.

Reviewer comment:

Line 206. Remove "was."
Reply:
We have removed the word "was." Please see the new line 202.

Reviewer comment:
Line 207. Replace "the" with "a."
Reply:
We have replaced the word "the" with "a." Please see the new line 204 .

Reviewer comment:
Line 208. Place a comma after "absorption."
Reply:
We have placed a comma after "absorption." Please see the new line 205.

Reviewer comment:
Line 209. Remove "the."
Reply:
We have removed the word "the." Please see the new line 206.

Reviewer comment:
Line 210. Remove "the."
Reply:
We have removed the word "the." Please see the new line 206.

Reviewer comment:
Line 211. Replace "has been required" with "is necessary."
Reply:
We have replaced the words "has been required" with "was necessary" according to correction by the professional English editing. Please see the new line 208.

Reviewer comment:
Line 212. Consider replacing this line with the following: "Hexaiodoplatinate reagent (HIPR) is a dark red substance whose color changes to yellow when mixed with sulfur compounds."
Reply:
We have replaced the explanation in line 212 to "Hexaiodoplatinate reagent (HIPR) is a

dark red substance whose color changes to yellow when mixed with sulfur compounds." Please see the new lines 209-210.

Reviewer comment:
Line 215. Remove "as described by previous reports."
Reply:
We have removed the words "as described by previous reports." Please see the new line 211.

Reviewer comment:
Line 218. What other "organic solvents" and "other reagents" are you referring to?
Reply:
We have inserted the name of organic solvent "methanol" after words "organic solvents." The words "other reagents" indicate "formic acid or heptafluorobutyric acid" in the sentence. We have added the reason to use them as "which does not give undesirable influences to MS system." Please see the new lines 215-216.

Reviewer comment:
Line 221. Replace "the established" with "this."
Reply:
We have replace the words "the established" with "this." Please see the new line 218.

Reviewer comment:
Line 222. Replace "increases" with "increased." Insert "the" before "aging." Remove ", proposing the production mechanism of it."
Reply:
We have replaced the word "increases" with "increased" and inserted the word "the" before "aging." We have removed the words ", proposing the production mechanism of it." Please see the new line 219.

Reviewer comment:
Line 224. Replace "can" with "could." Replace "the elucidation of" with "elucidating."
Reply:
We have replaced the word "can" with "could" and replaced the words "the elucidation of" with "elucidating." Please see the new line 221.

Reviewer comment:
Line 239. Replace "increases" with "increased."
Reply:
We have replaced the word "increases" with "increased." Please see the new line 237.

Reviewer comment:
Line 247. Remove "the."
Reply:
We have removed the word "the." Please see the new line 245.

Reviewer comment:
Line 248. Replace "up to" with "down."
Reply:
We have replaced the words "up to" with "to" according to correction by the professional English editing. Please see new line 245.

Reviewer comment:
Line 250. Remove the second "the."
Reply:
We have removed the second "the." Please see the new line 248.

Reviewer comment:
Line 254. What is the specific "preparation" referred to here?
Reply:
We have replace the word "preparation" with "AGE" because Figure 6C shows the change of cis/trans-S1PC in the preparation, aged garlic extract (AGE). The changes of those compounds are important to explain our hypothesis about increase of cis-form. Please see the new line 252.

Reviewer comment:
Line 256. Make "contents" singular.
Reply:
We have made "contents" singular. Please see the new line 253.

Reviewer comment:
Line 258. Replace "reaction from" with "of."

Reply:

We have replaced the words "reaction from" with "of." Please see the new line 256.

Reviewer comment:

Line 263. Replace "had become" with "became."

Reply:

We have replaced the words "had become" with "became." Please see the new line 261.

Reviewer comment:

Line 264. Remove the first "the."

Reply:

We have removed the first "the." Please seethe new line 261.

Reviewer comment:

Line 266. Insert "the" after "produce."

Reply:

We have inserted the word "the" after "produce." Please see the new line 263.

Reviewer comment:

Line 268. Remove "reaction" and "each."

Reply:

We have removed the words "reaction" and "each." We have rewritten “produced from trans-S1PC by……[18]. In their study, each of cis-S1PC” to “by isomerization of *trans*–S1PC during the aging period [18]. In their study, *cis*–S1PC and *trans*–S1PC were” Please see the new lines 265-266.

Reviewer comment:

Line 269. Remove "of." Replace "was" with "were."

Reply:

We have removed the word "of" and replaced the word "was" with "were." Please see new line 267.

Reviewer comment:

Line 270. Replace "dispended" with dispensed."

Reply:

We have replaced the word "dispended" with dispensed." Please see the new line 268.

Reviewer comment:

Line 272. Replace the second "in" with "of."

Reply:

We have replaced the second "in" with "of." Please see the new line 269.

Reviewer comment:

Line 275. Remove the first "the."

Reply:

We have removed the first "the." Please see the new line 272.

Reviewer comment:

Line 276. Insert "the" before "aging."

Reply:

We have inserted the word "the" before "aging." Please see the new line 273.

Reviewer comment:

Line 277. Remove the first "the" and "process."

Reply:

We have removed the first "the" and the word "process." Please see the new line 274.

Reviewer comment:

Line 281. Remove "and its containing."

Reply:

We have removed the words "and its containing." Please see the new line 278.

Reviewer comment:

Line 282. Remove "preparations." How are these important for maintenance of human health?

Reply:

We have removed the word "preparations." We have added the words "such as anti-cancer effects, antioxidant effect, neuroprotection, cholesterol reduction may be important for maintenance of human health [3, 9, 10]." after words "S1PC," to explain the "important for maintenance." Please see the new lines 278-280.

Reviewer comment:

Line 283. Remove "the."
Reply:
We have remove the word "the." Please see the new line 280.

Reviewer comment:
Line 284. Replace "the" with "its." Also, this sentence is misleading as it implies that good absorption and distribution equate to good pharmacological effects. This is not always the case. They can certainly aid in achieving good pharmacological activity. Please revise to make this aspect more clear.
Reply:
We have replaced the word "the" with "its." We have rewritten "the line 284" to "SAC possesses the important properties to exert its pharmacologic effects through absorption and distribution after consumption of its containing preparations." Please see the new lines 281-283.

Reviewer comment:
Line 285. Remove "a unique garlic preparation" as well as the second "in AGE."
Reply:
We have removed the words "a unique garlic preparation" and the second "in AGE. "Please see the new line 283.

Reviewer comment:
Line 292. Replace "The immunomodulatory effects of" with "Immunomodulation by." Otherwise you use the term "immunomodulatory effect" in quick succession.
Reply:
We have replaced the words "The immunomodulatory effects of" with "Immunomodulation by." Please see the new line 290.

Reviewer comment:
Line 293. Replace "have" with "has."
Reply:
We have replaced the word "have" with "has." Please see the new line 291.

Reviewer comment:
Line 296. Remove "the."
Reply:

We have removed the word "the."　Please see the new line 294.

Reviewer comment:
Line 297.　Remove "the."
Reply:
We have removed the word "the." Please see the new line 295.

Reviewer comment:
Line 301.　Remove the comma after "mediators."　Insert "In vitro studies revealed that" before "other."
Reply:
We have removed the comma after "mediators" and inserted the words "In vitro studies revealed that" before "other."　Please see the new line 299.

Reviewer comment:
Line 302.　Insert "including" before "DAS."　Replace the next two commas with dashes.
Reply:
We have inserted the number of compounds, such as "DAS (**16**), DADS (**17**) and DATS (**18**)", because they are different compounds. Please see the new line 300.

Reviewer comment:
Line 303.　Remove comma after "production" and insert "as well as."
Reply:
We have removed a comma after "production" and inserted the word　"as well as." Please see new line 302.

Reviewer comment:
Line 304.　Remove "in vitro studies."
Reply:
We have removed the words "in vitro studies." Please seethe new line 303.

Reviewer comment:
Line 309.　Remove "the."　Make "cell" plural.　Replace "under" with "during."
Reply:
We have removed the word "the", made "cell" plural and replaced the word "under" with

"during."　Please see the new line 308.

Reviewer comment:
Line 311.　Remove "the."
Reply:
We have removed the word "the." Please see the new line 311.

Reviewer comment:
Line 312.　Replace "factors" with "mediators."
Reply:
We have replaced the word "factors" with "mediators."Please see new line 311.

Reviewer comment:
Line 313.　Add a comma after "compounds."
Reply:
We have added a comma after "compounds." Please see the new line 312.

Reviewer comment:
Line 314.　Replace "study" with "studies."　Add a comma after "studies."
Reply:
We have replaced the word "study" with "studies" and added a comma after "studies." Please see the new line 313.

Reviewer comment:
Line 317.　Make "level" plural.
Reply:
We have made "level" plural. Please see the new line 316.

Reviewer comment:
Line 322.　Replace "The quantitative" with "Quantitative."
Reply:
We have replace the words "The quantitative" with "Quantitative." Please see the new line 321.

Reviewer comment:
Line 324.　Replace "and" with "to."

Reply:
We have replace the word "and" with "to." Please see the new line 323.

Reviewer comment:
Line 327. Replace the period after "Pax5" with a comma. Replace "Then the expression of Xbp1 mRNA is increased" with "thereby increasing the expression of Xbp1 mRNA."
Reply:
We have replace the period after "Pax5" with a comma. We have replace the words Replace "Then the expression of Xbp1 mRNA is increased" with "thereby increasing the expression of Xbp1 mRNA." Please see the new lines 326-327.

Reviewer comment:
Line 328. Revise the second sentence to read: "Xbp1 induces the expression of several genes." Revise the final sentence to read: "Illustration based on literature reports."
Reply:
We have revised the second sentence in the line 328 to "Xbp1 induces the expression of several genes" and the final sentence to "Illustration based on a literature reports." Please see the new lines 327-328.

Reviewer comment:
Line 337. Replace "defense in" with "of immune defense by."
Reply:
We have replaced the word "defense in" with "of immune defense by." Please see the new line 336.

Reviewer comment:
Line 344. Make "cell" plural.
Reply:
We have made "cell" plural. Please see the new line 343.

Reviewer comment:
Line 350. Replace "contribute to the human health" with "bolster human immunes responses."
Reply:
We have replaced the words "contribute to the human health" with "bolster human

immunes responses." Please see the new line 349.

Reviewer comment:
Line 354. Revise the first sentence to read as follows: "Life-style and diet have strong influences on human health."
Reply:
We have revise the first sentence in the line 354 to "Life-style and diet strongly influence human health." Please see the new line 353.

Reviewer comment:
Line 355. Make "contracts" singular. Omit "some."
Reply:
We have made "contracts" singular and omitted the word "some." Please see the new lines 353 and 354.

Reviewer comment:
Line 356. Replace "all over the world" with "world-wide."
Reply:
We have replaced the words "all over the world" with "world-wide." Please see the new line 355.

Reviewer comment:
Line 357. Replace "frequent problems of health management" with "frequently encountered health problems."
Reply:
We have replaced the words "frequent problems of health management" with "frequently encountered health problems." Please see the new line 356.

Reviewer comment:
Line 358. Make "effects" singular. Change "supplements" to "supplementation." Replace "have" with "has." Insert "numerous" before "clinical." Provide references to support this statement.
Reply:
We have made "effects" singular and changed the word "supplements" to "supplementation." We have replace the word "have" with "has" and inserted the word "numerous" before "clinical." We have added the reference [87]. Please see the new

lines 356-357.

Reviewer comment:
Line 360. Remove "total."
Reply:
We have removed the word "total." Please see the new line 359.

Reviewer comment:
Line 362. Place "dehydrated" before "raw."
Reply:
We have place the word "dehydrated" before "raw." Please see the new line 361.

Reviewer comment:
Line 366. Replace "8-week treatment" with "8 weeks of AGE treatment." Insert "observed" after "differences."
Reply:
We have replace the words "8-week treatment" with "8 weeks of AGE treatment", and finally rewritten to "8 weeks of AGE treatment with significant differences between." Please see the new line 365.

Reviewer comment:
Line 367. Remove "of AGE."
Reply:
We have not removed the words "of AGE" to avoid misunderstanding. Please see the new line 366.

Reviewer comment:
Line 369. Replace "a useful" with "an."
Reply:
We have replaced the words "a useful" with "an." Please see the new line 368.

Reviewer comment:
Line 372. Revise this line to read as follows: "performed using SHRS and the effect in the raw garlic group was noticeably stronger than those animals receiving AGE."
Reply:
We have rewritten the line 372 to "Comparison of the antihypertensive effects of raw

garlic and AGE on SHRs showed that the effects in the raw garlic group were noticeably stronger than those in animals receiving AGE" according to suggestion of reviewer and correction by professional English editing. Please see the new lines 370-372.

Reviewer comment:
Line 373. Insert "the" before "raw." Remove the first "an."
Reply:
We have inserted the word "the" before "raw" and removed the first "an." Please see the new line 372.

Reviewer comment:
Line 375. Insert "the" before "AGE."
Reply:
We have inserted the word "the" before "AGE." Please see the new line 374.

Reviewer comment:
Line 378. Replace "bioavailability of alliin" with "alliin's bioavailability."
Reply:
We have replaced the words "bioavailability of alliin" with " the bioavailability of alliin" according to reviewer comment and correction by professional English editing. Please see the new lines 377-378.

Reviewer comment:
Line 379. Revise the sentence beginning with "35S-Labeled" to read as follows: "85.5% of 35S-labeled allicin was excreted in the urine and feces of rats..."
Reply:
We have rewritten the sentence beginning with "35S-Labeled" to "85.5% of ^{35}S–Labeled allicin was excreted in urine and feces in rats [93],…." Please see the new line 379.

Reviewer comment:
Line 382. Replace "with the bioavailability almost 100%" with ", with a bioavailability of almost 100%."
Reply:
We have replaced the words "with the bioavailability almost 100%" with ", with a bioavailability of almost 100%." Please see the new line 381.

Reviewer comment:
Line 383. Make "profile" plural.
Reply:
We have made "profile" plural. Please see the new line 382.

Reviewer comment:
Line 385. Replace "5/6" with "using partially." Place a period after "rats." Remove "in which" and begin a new sentence with "Treatment."
Reply:
The "5/6 nephrectomized rats " is a name of hypertensive animal model and the quoted reference [94] use this words. Therefore we have not replaced the word "5/6" with "using partially." We have placed a period after "rats." We have removed the words "in which" and began a new sentence with "Treatment." Please see the new lines 384-385.

Reviewer comment:
Line 386. Remove "the." Insert "other indicators" after "as well as."
Reply:
We have removed the word "the" and inserted the words "other indicators" after "as well as." Please see the new line 386.

Reviewer comment:
Line 387. Replace "the" with "of."
Reply:
We have replaced the word "the" with "of." Please see the new line 386.

Reviewer comment:
Line 406. Insert "was' before "not." Make "control" plural.
Reply:
We have inserted the word "was' before "not" and made "control" plural. Please see the new line 405.

Reviewer comment:
Line 407. Replace the first "the" with "a." Remove the next two "the."
Reply:
We have replaced the first "the" with "a" and removed the next two "the." Finally, we rewritten “the line 407” to “Metabolomics analyses of plasma samples collected from

SHRs after completion of the treatment showed that plasma concentrations of 7 metabolites were affected by the treatment of S1PC (Table 1)" according to reviewer comment and correction by the professional English editing. Please see the new lines 406-407.

Reviewer comment:
Line 408. Remove "by using LC-MS method."
Reply:
We have removed the words "by using LC-MS method." Please see new line 406.

Reviewer comment:
Line 409. Remove "on four metabolic pathways."
Reply:
We have removed the words "on four metabolic pathways. "Please see the new line 407.

Reviewer comment:
Line 410. Replace "the" with "a."
Reply:
We have replaced the word "the" with "a." Please see the new line 408.

Reviewer comment:
Line 413. Remove "level." Remove "group of". Insert "group" after "treatment." Insert "the" after "to."
Reply:
We have removed the word "level" and the words "group of". We have inserted the word "group" after "treatment" and inserted the word "the" after "to." Finally, we have rewritten "the lines 413-414" to "]. Thus, the reduction of betaine in the S1PC treatment group may be partially related to the antihypertensive effect of S1PC" according to reviewer comment and correction by the professional English editing. Please see the new lines 410-411.

Reviewer comment:
Line 415. Insert "plasma" before "tryptophan." Make "level" plural. Remove "in plasma."
Reply:
We have inserted the word "plasma" before "tryptophan", made "level" plural and

removed the words "in plasma." Please see the new line 413.

Reviewer comment:
Line 416. Remove "the."
Reply:
We have removed the word "the." Please see the new line 414.

Reviewer comment:
Line 417. Make "phospholipids" singular. Make "concentration" plural. Replace "was" with "were." Replace "of" with "receiving."
Reply:
We made "phospholipids" singular and "concentration" plural. We have replaced the word "was" with "were" and the word "of" with "receiving." Finally, we have rewritten "the paragraph in the line 417 " to "concentrations of phospolipids were also observed in the group receiving S1PC treatment" according to reviewer comment and correction by the professional English editing. Please see the new line 415.

Reviewer comment:
Line 418. Remove the "and" before "glycine."
Reply:
There is no word "glycine" in the line 418. This word is in the line 420. The names of compounds indicate each metabolism pathway. We have rewritten as follows; "glycerolipid metabolism, tryptophan metabolism, and glycine metabolism, serine and threonine metabolism," Please see the new lines 418-419.

Reviewer comment:
Line 428. Remove "in liver and kidney." Remove "into urine and bile." Place a period after "excretion." Replace "the" with "a."
Reply:
We have removed the words "in liver and kidney" and "into urine and bile", and placed a period after "excretion" and replaced the word "the" with "a." Please see the new lines 426-427 .

Reviewer comment:
Line 431. Remove "the."
Reply:

We have removed the word "the." Please see the new line 430.

Reviewer comment:
Line 433. Remove "these compounds." Make "rodents" singular.
Reply:
We have removed the words "these compounds" and made the word "rodents" singular. Please see the new lines 431 and 432.

Reviewer comment:
Line 434. Remove "maybe."
Reply:
We have removed the word "maybe." Please see the new line 432.

Reviewer comment:
Line 436. Insert "the" before "oral."
Reply:
We have inserted the word "the" before "oral." Please see the new line 434.

Reviewer comment:
Line 438. Insert "a" before "relatively." Replace "bioavailabilities" with "bioavailability." Place a colon after "bioavailability."
Reply:
We have inserted the word "a" before "relatively", replaced the word "bioavailabilities" with "bioavailability" and placed a colon after "bioavailability." Please see the new line 437.

Reviewer comment:
Line 439. Remove "the interesting result."
Reply:
We have removed the words "the interesting result." Please see the new line 439.

Reviewer comment:
Line 442. Remove "its." Replace "of" with "ranging from."
Reply:
We have removed the word "its" and replaced the word "of" with "ranging from." We have added the word “between” before the word “ranging.” Please see the new line 440.

Reviewer comment:

Line 443. Remove "the." Make "concentration" plural.

Reply:

We have removed the word "the" and made "concentration" plural. Please see the new line 441.

Reviewer comment:

Line 447. Remove "the."

Reply:

We have removed the word "the." Please see the new line 445.

Reviewer comment:

Line 448. Remove the second "the."

Reply:

We have removed the second "the." Please see the new line 447.

Reviewer comment:

Line 451. Remove "metabolism."

Reply:

We have removed the word "metabolism." Please see the new line 449.

Reviewer comment:

Line 452. Place a comma after "species."

Reply:

We have placed a comma after "species." Please see the new line 450.

Reviewer comment:

Line 453. Replace "unchanged" with "parent." Replace the second "the" with "an."

Reply:

We have replaced the word "unchanged" with "parent" and the second "the" with "an." Finally, we have rewritten "the paragraph containing these words" to "*N*–acetylated metabolites being 1/2 to 1/3 of those of the parent forms. Additionally, an *in vitro* metabolism study" according to reviewer comment and correction by professional English editing. Please see the new line 451.

Reviewer comment:

Line 459. Replace "a small extent" with "a much smaller extent." Begin the second sentence with "The authors concluded that the kidney plays..."

Line 460. Insert "unchanged" before "SAC." End the sentence with "dogs." Omit everything going forward from "indicating that..."

Reply:

We have replaced the words "a small extent" with "a much smaller extent" and began the second sentence with the "The authors concluded that the kidney plays..."

We have inserted the word "unchanged" before "SAC." We have ended the sentence with the word "dogs" and omitted the words "indicating that SAC and S1PC were excreted into the urine as the unchanged forms after undergoing the deacetylation in the kidney." Please see the new lines .

Finally, we have rewritten the sentences "By contrast, …… in the kidney" in the lines 458-461 to the sentences "However, the *N*–acetylated metabolites of SAC and S1PC were excreted in the urine of dogs at much lower levels because kidney of dogs how extremely high deacetylation activities for *N*–acetylated metabolites [102]. The authors concluded that the kidney plays a critical role in the elimination of SAC and S1PC through renal excretion, reabsorption, and metabolism" according to reviewer comment and correction by the professional English editing. Please see the new lines 457-459.

Reviewer comment:

Line 465. Insert "a" before "concern."

Reply:

We have inserted the word "a" before "concern." Please see the new line 463.

Reviewer comment:

Line 475. Consider utilizing the following reference to summarize the clinical herb-drug interaction studies conducted with garlic supplements: "Gurley BJ, Fifer KE, Gardner Z. Pharmacokinetic herb-drug interactions (Part 2): drug interactions involving popular botanical dietary supplements and their clinical relevance. Planta Medica, 2012;78:1490-1514."

Reply:

We have added the sentence with the reference " Gurley et al. reviewed the findings from a number of studies for the evaluation of the drug interaction potential of garlic supplements *in vitro* and *in vivo*, concluding that commercially available garlic products, including AGE, have only a limited potential to produce clinically important herb-drug

interactions [103]." after the end of Line 475 according to reviewer’s comment. Additionally, the reference " Planta Medica, 2012;78:1490-1514" was added to the citation section as the reference number 103. Please see the new line 476, and the lines 771-773 in citation.

Reviewer comment:
Line 486. Consider revising the first sentence of the Conclusion to read as follows: "Since its identification in garlic more than 50 years ago, few reports concerning the biological properties of S1PC have appeared in the medical literature. Recent studies, however, have revealed a host of noteworthy pharmacological effects linked to S1PC."
Reply:
We have the first sentence with the sentence "Since its identification in garlic more than 50 years ago, few reports concerning the biological properties of S1PC have emerged in medical literature. Recent studies, however, have revealed a host of noteworthy pharmacological effects linked to S1PC" according to reviewer comment and correction by professional English editing. Please see the new lines 487-489.

Reviewer comment:
Line 489. Insert "both" after "possess."
Reply:
We have inserted the word "both" after "possess." Please see the new line 491.

Reviewer comment:
Line 491. Remove "the." Insert "experimental" before "animal."
Reply:
We have removed the word "the" and inserted the word "experimental" before "animal." Please see the new lines 492 and 493.

Reviewer comment:
Line 492. Remove "experimental."
Reply:
We have removed the word "experimental." Please see the new line 493.

Reviewer comment:
Line 493. Make "profile" plural. Replace "the high" with "excellent." Replace “long-lasting blood concentration” with “long elimination half-lives.”

Reply:

We have made "profile" plural, and replaced the words "the high" with "excellent," the words "long-lasting blood concentration" with "long elimination half-lives." Finally, we have rewritten “the paragraph containing these words” to “pharmacokinetic profiles in rats and dogs with the excellent bioavailability (88–100%), and long elimination half–lives,” according to reviewer comment and correction by the professional English editing. Please see the new line 494-495.

Reviewer comment:

Line 494. Replace "are" with "may be." Remove "properties of excellent oral absorption and."

Reply:

We have replaced the word "are" with "may be" and removed the words "properties of excellent oral absorption and." Please see the new line 495.

Reviewer comment:

Line 496. Remove the second "the."

Reply:

We have removed the second "the." Please see the new line 497.

Reviewer comment:

Line 497. Replace "suggested" with "suggest."

Reply:

We have replaced the word "suggested" with "suggest." Please see the new line 498.

Reviewer comment:

Line 498. Insert "the" before "prevention." Remove "the" at the end of the line.

Reply:

We have inserted the word "the" before "prevention" and removed the word "the" at the end of the line. Please see the new line 499.

Reviewer comment:

Line 499. Insert "illnesses" after "lifestyle-related."

Reply:

We have inserted the word "illnesses" after "lifestyle-related." Please see the new line 499.

****

Furthermore, we have revised and corrected several words, expressions and phrases according to correction by professional English editing as follows:

1. We have inserted the word "a" in new line 12 corresponding to the former line 12.
2. We have inserted the word "the" after "since" in new line 13 corresponding to the former line 13.
3. We have replaced semicolon to period after "1960's," and began the new sentence with "However", and replaced "concerning" to "regarding" in new line 14 corresponding to the former line 14.
4. We have removed "a" before "trace", replaced "content" with "concentration", inserted "almost" after "increased" in new line 17 corresponding to the former line 17.
5. We have rewritten "similar to that of SAC through the aging" to "similar of SAC through aging" in new line 18 corresponding to the former lines 17-18.
6. We have replaced "had" o "showed" in new line 18 corresponding to the former line 18.
7. We have removed "showed the ability to" in new line 18 corresponding to the former line 19.
8. We have added "oral" after "administration" in new line 20 corresponding to the former line 20.
9. We have removed "oral" before "bioavailability" in new line 21 corresponding to the former line 21.
10. We have replaced "like" with "similar to" in new line 23 corresponding to the former line 24.
11. We have inserted comma after "activities" and replaced "the" with "a" in new line 22 corresponding to the former line 22.
12. We have replaced "In this review, we highlight some results concerning the chemical synthesis and biosynthesis, the production mechanism in garlic preparation, the analytical procedure, the biological and pharmacologic effects, the pharmacokinetics and safety of S1PC and discuss its potential medicinal value" to "In this review, we highlight some results from recent studies on S1PC and discuss the potential medicinal value of S1PC" in new lines 24-25 corresponding to the former lines 24-27.
13. We have replaced "those" with "these" in new line 32 corresponding to the former line 34.

14. We have replaced "has" with "had", removed "the" before "medical," and replaced "of" with "against" in new line 33 corresponding to the former line 35.
15. We have replaced "there were no other useful medicinal supplies" with "other medicinal supplies were limited" in new line 34 corresponding to the former line 36.
16. We have removed "Even now" and added "still" after "use" in new line 35 corresponding to the former line 37.
17. We have replaced "preparations to obtain the beneficial effects for maintaining their health because there has been a lot of scientific reports that indicate the beneficial effects of garlic," with "as several scientific studies have reported its following properties; antioxidative," in new line 35-36 corresponding to the former line 37-39.
18. we have added ", and" after "anti-alzheimer's," and added "disease" after "anti-alzheimer's," in new line 37 corresponding to the former line 40.
19. We have replaced "Before modern times" with "Earlier," in new line 37 corresponding to the former line 40.
20. We have added "the" after "noteworthy" and replaced "finding" with "discovery" in new line 40 corresponding to the former line 43.
21. We have replaced "the chemistry of garlic" with "the studies on chemical properies" in new line 41 corresponding to the former line 44.
22. We have replaced "scientists" with "studies" in new line 42 corresponding to the former line 45.
23. We have removed "the" before "evaluation" in new line 43 corresponding to the former line 45.
24. We have replaced "those" with "these" in new line 42 corresponding to the former line 46.
25. we have replaced "concerning" with "regarding" in new line 44 corresponding to the former line 47.
26. We have replaced "presented" with "emerged" in new line 46 corresponding to the former line 49.
27. We have removed "the" in new line 47corresponding to the former line 50.
28. We have replaced "like" with "such as" and "as to whether" with "regarding" in new line 48 corresponding to the former line 51.
29. We have replaced "is" with "being" and inserted "beneficial" after "component" in new line 49 corresponding to the former line 52.
30. We have removed "the" after "hydrophilic" in new line 50 corresponding to the former line 53.
31. We have replaced "those" with "these" in new line 53 corresponding to the former

line 56.

32. we have removed “the” before “various” in new line 54 corresponding to the former line 57.
33. We have replaced “anticancer effect, antioxidant effect, cholesterol–lowering effect, anti–hepatotoxic effect and neuroprotective effect” with “anticancer, antioxidant, cholesterol–lowering, anti–hepatotoxic, and neuroprotective effects” in new line 55 corresponding to the former line 58.
34. We have replaced “to exist” with “to present” in new line 58 corresponding to the former line 61.
35. We have replaced “were” with “have been” in new line 58 corresponding to the former line 62.
36. We have replaced “that” with “which” in new line 60 corresponding to the former line 64.
37. We have inserted “the” before “lachrymatory” in new line 61 corresponding to the former line 64.
38. We have inserted comma after “[18, 19]” and “it” after “and” in new line 63 corresponding to the former line 66.
39. we have inserted “in” after “preparetions” in new line 64 corresponding to the former line 67
40. We have replaced “scientists focused on the studies of natural products concerning the concomitant use of food/supplements and medical agents, to assess their effectiveness and safety, and found that the” with “studies assessing the effectiveness and safety of natural products as food/supplements and medical agents showed that their” in new lines 65-66 corresponding to the former line 68-70.
41. We have removed “the” before “pharmacokinetic” in new line 67 corresponding to the former line 71.
42. We have removed “the” before “biological”, inserted “the” before “concentration”, and replaced “in” with “of” in new line 68 corresponding to the former lines 71-72.
43. We have replaced “and has” with “with” in new line 70 corresponding to the former line 74.
44. We have made “activities” singular in new line 71 corresponding to the former line 75.
45. We have removed “the” before “recent” in new line 73 corresponding to the former line 77.
46. We have replaced “compound [30, 31]. Considering the fact that the content of this compound in onion was less than 0.2% [16] and also several means of purification

steps were necessary to obtain it from the raw material plants, its organic synthesis method has been made," with "compound, a biologically active compound that is beneficial for human health [5] (pp. 60–99, pp. 107–132, pp. 282–297), [16, 17, 30, 31]. The content of this compound in onion is less than 0.2% [16], and its purification is difficult because many compounds with similar chemical characteristics exist in onion. Therefore, its organic synthesis method has been investigated to reveal sulfur chemistry in onion and other *Allium* vegetables, including the preparation of S1PC as an intermediate of S1PCSO [14–17]" in new lines 80-85 corresponding to the former line 84-86.

47. We have inserted "the" after "Since," and "of" after "S1PC," and comma after "S1PC" in new line 87 corresponding to the former line 89.
48. We have replaced "middle of" with "mid" and "showed the" with "reported a" in new line 93 corresponding to the former line 95.
49. We have replaced "gave both *cis*– and *trans*–form S1PC in the almost equal ratio, and *cis*–form crystalline was mainly" with "yieled both *cis*– and *trans*–S1PC in almost equal amounts, and the *cis*–form was mainly" in new line 95 corresponding to the former line 97-98.
50. We have inserted "the" before "concentrated" in new line 96 corresponding to the former line 98.
51. We have replaced "e.g." with "such as" in new line 97 corresponding to the former line 99.
52. We have made "condition" plural and made "induces" singular in new line 99 corresponding to the former line 101.
53. We have replaced "of" with "for" in new line 104 corresponding to the former line 106.
54. We have removed "the" before "reversible," and replaced "in" with "under," and made "condition" plural in new line 112 corresponding to the former line 115.
55. We have replaced "the" with "a" in new line 113 corresponding to the former line 116.
56. We have replaced "study must be made to accomplish the sufficient" with "study must be made to accomplish the sufficient" in new line 115 corresponding to the former lines 117-118.
57. We have replaced "briefly from" with "based on" in new line 119 corresponding to the former line 122.
58. We have inserted the compound number "(**7**)" after "(isoalliin)" in new line 133 corresponding to the former line 136.

59. We have inserted “the” after “In” in new line 133 corresponding to the former line 137.
60. We have inserted the compound number “(**7**)” after “Isoalliin” in new line 139 corresponding to the former line 142.
61. We have replaced “from literatures” with “previous reports” in new line 141 corresponding to the former line 144.
62. We have made “name” plural, inserted “the” before “numbered,” and replaced “is” with “are” in new line 142 corresponding to the former line 145.
63. We have inserted the compound number “(**10**)” after “GSAC,” in new line 145 corresponding to the former line 147.
64. We have inserted the compound number “(**7**)” after “Isoalliin” in new line 139 corresponding to the former line 148.
65. We have replaced semicolon with period after “[18, 19]” and began new sentence with “However,” in new line 145 corresponding to the former line 148.
66. We have replaced “studied the involvement of” with “showed that” in new line 148 corresponding to the former lines 151-152.
67. We have replaced “in the biosynthesis of alliin in garlic, which was shown to catalyze the highly stereoselective *S*-oxygenation reaction” with “catalyze the highly stereoselective *S*-oxygenation reaction in the biosynthesis of alliin in garlic” in new lines 149-150 corresponding to the former lines 152-153.
68. We have inserted “the” before “S-oxidation” in new line 151 corresponding to the former line 155.
69. We have added the compound number “(6)” after “alliin” in new line 156 corresponding to the former line 159.
70. We have inserted “, who identified” after “Yoshimoto et al.” in new line 157 corresponding to the former line 160.
71. We have inserted “the” before “alliin,” comma after “content,” compound number “(**11**)” and” content” after “trans-GS1PC,” and “reaction” after “S-oxygenetion” in new line 160 corresponding to the former lines 162-163.
72. We have replaced “increase” with “contents increased” in new line 163 corresponding to the former line 166.
73. We have replaced “was drawn based on literature” with “is based on mechanisms published in previous reports” in new line 164 corresponding to the former lines 166-167.
74. We have inserted “the” before “alliin” in new line 166 corresponding to the former line 168.

75. We have made “name” plural, inserted “the” before “numbered,” and replaced “is” with “are” in new line 167 corresponding to the former line 169.
76. We have removed “also” in new line 169 corresponding to the former line 171.
77. We have replaced “by” with “in” in new line 180 corresponding to the former line 182.
78. We have replaced “by” with “in” in new line 181 corresponding to the former line 183.
79. We have removed “an” before “outstanding” and made “ability” plural in new line 187 corresponding to the former line 190.
80. We have removed “the” before “pyrolysis” and “formation” in new line 190 corresponding to the former line 193.
81. We have replaced “because” with “as,” made “temperature” plural, and replaced “an” with “the” in new line 191 corresponding to the former lines 194-195.
82. We have replaced “a” with “the” before “GC-column” and “gave” with “resulted in” in new line 192 corresponding to the former lines 195-196.
83. We have removed “On the other hand,” before “high” and began new sentence with “High” in new line 194 corresponding to the former line 197.
84. We have replaced “without causing” with “to avoid” in new line 196 corresponding to the former line 199.
85. We have removed “by” before “using” in new line 197 corresponding to the former line 200.
86. We have replaced semicolon with period after “products” and began new sentence with “However” in new line 199 corresponding to the former line 202.
87. We have replaced “occur due to ” with “because of the use of” in new line 200 corresponding to the former line 203.
88. We have replaced semicolon with period after “absorption” and began new sentence with “However” in new line 201 corresponding to the former line 204.
89. We have replaced “has been” with “was” in new line 208 corresponding to the former line 211.
90. We have inserted “an” before “organic” in new line 215 corresponding to the former line 218.
91. We have replaced “they found that cis-form S1PC exist in AGE” with “cis-form S1PC was shown to exist in AGE” in new lines 218-219 corresponding to the former line 221.
92. We have inserted “a” before “previous” in new line 227 corresponding to the former line 230.

93. We have replaced “its” with “their” in new line 228 corresponding to the former line 231.
94. We have made “time” and “name” plural, inserted “the” before “numbered,” and replaced “is” with “are” in new line 229 corresponding to the former lines 231-232.
95. We have removed “was” before “decreased” in new line 236 corresponding to the former line 238.
96. We have removed “the” before “hydrolysis” in new line 238 corresponding to the former line 240.
97. We have replaced “completely finished” with “complete” in new line 239 corresponding to the former line 241.
98. We have replaced “varies” with “varied” in new line 240 corresponding to the former line 243.
99. We have replaced “relating” with “related” in new line 242 corresponding to the former line 244.
100. We have replaced “content of GS1PC, a precursor of S1PC, in fresh garlic” with “content of GS1P in fresh garlic” in new lines 242-243 corresponding to the former line 245.
101. We have removed “was” in new line 244 corresponding to the former line 247.
102. We have added “the” before “GSAC” in new line 246 corresponding to the former line 249.
103. We have added “the” before “aging” in new line 255 corresponding to the former line 257.
104. We have replaced “was” with “is” and added “reports” after “literatures” in new line 257 corresponding to the former line 259.
105. We have replaced “existence” with “presence” in new line 261 corresponding to the former line 264.
106. We have replaced semicolon with period after “[32]” and began new sentence with “However,” in new line 263 corresponding to the former line 265.
107. We have replaced “from trans-S1PC by isomerization reaction” with “by isomerization of trans-S1PC” and inserted “the” before “aging” in new line 265 corresponding to the former line 268.
108. We have removed “at” before “pH” in new line 267 corresponding to the former line 270.
109. We have replaced “by” with “using” in new line 268 corresponding to the former line 271.
110. We have removed “were” before “gradually” and replaced “became” with “were” in

new line 270 corresponding to the former lines 272-273.

111. We have removed “the” before “isomerization” in new line 271 corresponding to the former line 273.
112. We have removed “phenomenon” in new line 273 corresponding to the former line 276.
113. We have replaced “which prodeces” with “producing” in new line 274 corresponding to the former line 277.
114. We have replaced “on S1PC regarding its biological effects, pharmacokinetics and safety” with “regarding the biological effects, pharmacokinetics and safety of S1PC ” in new lines 284-285 corresponding to the former lines 286-287.
115. WE have replaced “thought” with “speculated” in new line 290 corresponding to the former line 292.
116. We have removed “the” before “enhanced” in new line 296 corresponding to the former line 299.
117. We have inserted “, which are” before “major” in new line 300 corresponding to the former line 302.
118. We have replaced “On the other hand” with “On contrary” in new line 304 corresponding to the former line 305.
119. We have inserted “levels” after “TNF-α” in new line 308 corresponding to the former line 309.
120. We have replaced “possessed” with “showed” in new line 309 corresponding to the former line 310.
121. We have removed “a” before “low” in new line 310 corresponding to the former line 311.
122. We have replaced “by” with “using” and inserted “a” before “Student’s” in new line 325 corresponding to the former lines 325-326.
123. We have replaced “productin” with “production” in new line 342 corresponding to the former line 343.
124. We have made “macrophage” plural in new line 343 corresponding to the former line 344.
125. We have removed “the” before “induction” in new line 344 corresponding to the former line 345.
126. We have made “disease” plural in new line 355 corresponding to the former line 357.
127. We have replaced “whereas” with “although” in new line 369 corresponding to the former line 370.

128. We have removed “their” before “detailed” in new line 376 corresponding to the former line 377.
129. We have added “the” after “that” in new line 377 corresponding to the former line 378.
130. we have replaced “On the other hand” with “On contrary” in new line 380 corresponding to the former line 381.
131. We have replaced “that” with “those observed” in new line 382 corresponding to the former line 383.
132. We have made “metabolites” singular and inserted “levels” before “in” in new line 388 corresponding to the former line 389.
133. We have replaced “from” with “by referring to a previous study” in new line 390 corresponding to the former line 391.
134. We have added “Waltham, MA, USA” after “Scientific” in new line 393 corresponding to the former line 394.
135. We have removed “Waltham, MA, USA” after “Scientific,” in new line 394 corresponding to the former line 395.
136. We have replaced “to which” with “which were orally administrated” in new lines 401-402 corresponding to the former line 402.
137. We have removed “were orally administrated” after “(6.5 mg/kg)” in new line 403 corresponding to the former line 404.
138. We have added “the” after “from” in new line 405 corresponding to the former line 406.
139. We have removed “Because” before “betaine” and began new sentence “Betain” in new line 409 corresponding to the former line 411.
140. We have replaced comma with period after “[95]” and began new sentence “Thus” in new line 410 corresponding to the former line 412.
141. We have inserted “be” after “may” and removed “be” after “treatment” in new line 411 corresponding to the former line 413.
142. We have inserted “the” before “blood” and removed “the” before “alteration” in new line 412 corresponding to the former lines 414-415.
143. We have replaced “produces its ” with “reduces the” in new line 417 corresponding to the former line 419.
144. We have replaced “profiled. These” with “profiles, which” in new line 425 corresponding to the former line 426.
145. We have replaced “present in” with “from” in new line 429 corresponding to the former line 430.

146. We have replaced “were orally absorbed from” with “were absorbed in the” in new line 431 corresponding to the former line 433.
147. We have removed “in rats” after cycloalliin and inserted “in rats” after “onion” in new line 436 corresponding to the former line 437.
148. We have replaced “of” with “between” after “ranging” in new line 440 corresponding to the former line 442.
149. We have replaced reference number “[102]” with “[12]” in new line 442 corresponding to the former line 444.
150. We have replaced “had” with “showed” in new line 445 corresponding to the former line 447.
151. We have replaced “that of” with “of those” in new line 451 corresponding to the former line 452.
152. We have replaced “study of metabolism” with “metabolism study” in new line 452 corresponding to the former line 454.
153. We have replaced “By contrast” with “However” in new line 456 corresponding to the former line 458.
154. We have replaced “to a small extent. Authors concluded that kidney had a critical role in the elimination of SAC and S1PC in dogs, indicating that SAC and S1PC were excreted into the urine as the unchanged forms after undergoing the deacetylation in the kidney” with “dogs at much lower levels because kidney of dogs how extremely high deacetylation activities for *N*–acetylated metabolites [102]. The authors concluded that the kidney plays a critical role in the elimination of SAC and S1PC through renal excretion, reabsorption, and metabolism” in new lines 457-459 corresponding to the former lines 459-461.
155. We have replaced “finding on” with “studies regarding” in new line 460 corresponding to the former line 462.
156. We have replaced “dosing” before “oral” and “preparations was increased” with “preparations increased” in new line 462 corresponding to the former line 464.
157. We have removed “the” before “concomitant” in new line 464 corresponding to the former line 466.
158. We have replaced “cause the enhancement” with “enhance” in new line 465 corresponding to the former line 466.
159. We have removed “the” before “enzymatic” in new line 466 corresponding to the former line 468.
160. We have removed “the” before “IC_{50}” in new line 468 corresponding to the former line 470.

161. We have replaced “On the other hand” with “On the contrary,” replaced the position of SMC and SAC, replaced “had” with “showed,” and made “effect” plural in new line 469 corresponding to the former lines 470-471.
162. We have inserted comma after “isomers” and “mM,” replaced “except” with “in,” and replaced semicolon with colon in new line 470 corresponding to the former line 472.
163. We have removed “even at 1mM” and inserted period after 31% in new line 471 corresponding to the former line 473.
164. We have replaced “due to” with “through” in new line 472 corresponding to the former line 474.
165. We have replaced “excretes” with “excretion levels” in new line 478 corresponding to the former line 477.
